# Buprenorphine Utilization and Prescribing Among New Jersey Medicaid Beneficiaries After Adoption of Initiatives Designed to Improve Treatment Access

**DOI:** 10.1001/jamanetworkopen.2023.12030

**Published:** 2023-05-05

**Authors:** Peter Treitler, Molly Nowels, Hillary Samples, Stephen Crystal

**Affiliations:** 1 Institute for Health, Health Care Policy and Aging Research, Rutgers University, New Brunswick, New Jersey; 2 School of Social Work, Rutgers University, New Brunswick, New Jersey; 3 School of Public Health, Rutgers University, Piscataway, New Jersey

## Abstract

**Importance:**

Buprenorphine is underutilized as a treatment for opioid use disorder (OUD); state policies may improve buprenorphine access and utilization.

**Objective:**

To assess buprenorphine prescribing trends following New Jersey Medicaid initiatives designed to improve access.

**Design, Setting, and Participants:**

This cross-sectional interrupted time series analysis included New Jersey Medicaid beneficiaries who were prescribed buprenorphine and had 12 months continuous Medicaid enrollment, OUD diagnosis, and no Medicare dual eligibility, as well as physician or advanced practitioners who prescribed buprenorphine to Medicaid beneficiaries. The study used Medicaid claims data from 2017 to 2021.

**Exposure:**

Implementation of New Jersey Medicaid initiatives in 2019 that removed prior authorizations, increased reimbursement for office-based OUD treatment, and established regional Centers of Excellence.

**Main Outcomes and Measures:**

Rate of buprenorphine receipt per 1000 beneficiaries with OUD; percentage of new buprenorphine episodes lasting at least 180 days; buprenorphine prescribing rate per 1000 Medicaid prescribers, overall and by specialty.

**Results:**

Of 101 423 Medicaid beneficiaries (mean [SD] age, 41.0 [11.6] years; 54 726 [54.0%] male; 30 071 [29.6%] Black, 10 143 [10.0%] Hispanic, and 51 238 [50.5%] White), 20 090 filled at least 1 prescription for buprenorphine from 1788 prescribers. Policy implementation was associated with an inflection point in buprenorphine prescribing trend; after implementation, the trend increased by 36%, from 1.29 (95% CI, 1.02-1.56) prescriptions per 1000 beneficiaries with OUD to 1.76 (95% CI, 1.46-2.06) prescriptions per 1000 beneficiaries with OUD. Among beneficiaries with new buprenorphine episodes, the percentage retained for at least 180 days was stable before and after initiatives were implemented. The initiatives were associated with an increase in the growth rate of buprenorphine prescribers (0.43 per 1000 prescribers; 95% CI, 0.34 to 0.51 per 1000 prescribers). Trends were similar across specialties, but increases were most pronounced among primary care and emergency medicine physicians (eg, primary care: 0.42 per 1000 prescribers; 95% CI, 0.32-0.53 per 1000 prescribers). Advanced practitioners accounted for a growing percentage of buprenorphine prescribers, with a monthly increase of 0.42 per 1000 prescribers (95% CI, 0.32-0.52 per 1000 prescribers). A secondary analysis to test for changes associated with non–state-specific secular trends in prescribing found that quarterly trends in buprenorphine prescriptions increased in New Jersey relative to all other states following initiative implementation.

**Conclusions and Relevance:**

In this cross-sectional study of state-level New Jersey Medicaid initiatives designed to expand buprenorphine access, implementation was associated with an upward trend in buprenorphine prescribing and receipt. No change was observed in the percentage of new buprenorphine treatment episodes lasting 180 or more days, indicating that retention remains a challenge. Findings support implementation of similar initiatives but highlight the need for efforts to support long-term retention.

## Key Points

**Question:**

Were initiatives aimed at improving access to buprenorphine treatment in New Jersey Medicaid associated with increases in buprenorphine utilization and prescribing?

**Findings:**

This cross-sectional study of 20 090 Medicaid beneficiaries found that the New Jersey Medicaid initiatives were associated with significant increases in buprenorphine prescribing at the clinician level and in buprenorphine receipt for patients with opioid use disorder but no increase in the percentage of new buprenorphine treatment episodes lasting 180 or more days.

**Meaning:**

Findings suggest that policies and programs like the New Jersey Medicaid initiatives expanded buprenorphine treatment availability and access but highlight the need for further efforts to support long-term prescribing and retention.

## Teaser

This cross-sectional study assesses buprenorphine prescribing trends after the implementation of initiatives in New Jersey Medicaid designed to improve access.

## Introduction

Expanding access to evidence-based treatments for opioid use disorder (OUD) is a major policy priority in addressing the ongoing overdose epidemic.^1^ Yet, despite strong evidence demonstrating their effectiveness, medications for OUD (MOUDs) remain underutilized.^2^ Nationally, less than 30% of individuals who could potentially benefit from treatment are estimated to receive MOUDs,^3^ and among Medicaid beneficiaries diagnosed with OUD in 11 states in 2018, just 57% received MOUDs.^4^ Many efforts to increase access to MOUDs have focused on buprenorphine, which carries low risk of overdose and can be prescribed in any health care setting by appropriately licensed clinicians. However, the vast majority of clinicians do not prescribe buprenorphine,^5,6^ including less than 5% of primary care practitioners (PCPs).^5,7,8,9^

Key barriers to buprenorphine prescribing include concerns about the potential for greater Drug Enforcement Agency scrutiny; insufficient training and expertise regarding a patient population and treatment with high perceived complexity; low reimbursement; stigma; insufficient referral options for more intensive treatment; and concerns about diversion.^10,11,12,13,14^ These barriers may be amplified for the Medicaid population, as clinicians are less likely to accept Medicaid than other payment sources due to generally lower reimbursement rates.^15,16^ With limited access to MOUDs and an elevated burden of OUD,^17^ Medicaid patients represent a priority group for states, payers, and health systems aiming to increase buprenorphine utilization.

In 2019, New Jersey Medicaid implemented the MATrx model, which included initiatives designed to reduce barriers to buprenorphine prescribing in the Medicaid program and support clinicians in establishing or expanding capacity to deliver office-based addiction treatment. Under this model, Medicaid prohibited managed care organizations from requiring prior authorizations for MOUDs,^18^ to which most New Jersey Medicaid enrollees were subject before 2019.^19^ Medicaid also implemented the Office Based Addiction Treatment (OBAT) program, enhancing reimbursement for office-based MOUD visits in clinics offering patient navigation.^18^ The program further created new reimbursement codes for patient navigation in OBATs, which could be provided by nurses, social workers, medical assistants, or others who meet training and experiential requirements.^20^ Medicaid established 2 regional Medication for Addiction Treatment (MAT) Centers of Excellence (COEs), which provide training, consultation, and technical assistance to MOUD prescribers around the state.^21,22^ The MATrx model emphasized coordination across OBAT programs, COEs, and comprehensive care settings, such as federally qualified health centers.^23^ Together, these initiatives were intended to remove barriers and support practitioners in prescribing buprenorphine, with a focus on engaging PCPs and other general practitioners in treating OUD.

As states, payers, and other entities implement initiatives to expand access to MOUDs, there is a need to study their outcomes and whether intended goals were achieved to inform policies and practices. This study aims to assess whether the New Jersey Medicaid initiatives were associated with changes in buprenorphine receipt trends among Medicaid beneficiaries and buprenorphine prescribing among clinicians treating Medicaid patients. Specifically, this study examines the association of the initiatives with (1) buprenorphine receipt among patients with OUD; (2) percentage of new buprenorphine patients retained for 180 or more days; and (3) buprenorphine prescribing by clinician type and specialty.

## Methods

Study procedures were approved by the Rutgers institutional review board. Informed consent was waived because the study used deidentified secondary data collected for nonresearch purposes. This study follows the Strengthening the Reporting of Observational Studies in Epidemiology (STROBE) reporting guidelines for cross-sectional studies.

### Data and Sample

We analyzed New Jersey Medicaid claims from 2017 to 2021. For patient-level analyses, Medicaid beneficiaries were included in the sample if they were aged 18 to 64 years, had continuous Medicaid enrollment in the current month and prior 12 months, were not dually eligible for Medicare throughout this period, and had an OUD diagnosis on at least 1 claim during this period. Clinician-level analyses included all physicians and advanced practitioners (nurse practitioners, physician’s assistants) who prescribed any medication to Medicaid beneficiaries.

### Measures

#### Buprenorphine Receipt and Retention

We calculated the number of patients per 1000 beneficiaries with OUD who were prescribed buprenorphine in each month, including only buprenorphine formulations indicated for OUD (eTable 1 in Supplement 1). We used rates per 1000 instead of percentages to facilitate interpretation of model estimates.

To assess the association of initiatives with treatment duration, we also calculated the monthly percentage of new buprenorphine episodes lasting at least 180 days among beneficiaries continuously enrolled for at least 6 months after the start of a new episode. New episodes were defined as a buprenorphine fill following at least 30 days without buprenorphine supply. Among patients with new buprenorphine episodes in each month, 180-day retention was defined as buprenorphine supply on at least 144 of 180 days (80%) after the new prescription fill date, and supply on at least 1 day in days 150 to 180. Patient-level analyses adjusted for monthly percentages of individuals in the denominator with White race, male sex, comorbid psychiatric diagnosis, and comorbid non-OUD substance use disorder as well as mean age.

#### Buprenorphine Prescribing and Clinician Type

We identified clinician types and specialties using National Provider Identification (NPI) numbers on prescription claims, linked to the National Plan and Provider Enumeration System NPI file,^24^ the National Uniform Claim Committee Health Care Provider Taxonomy code set,^25^ and clinician information contained in Medicaid claims (eTable 2 in Supplement 1). To assess whether policy implementation was associated with a broadened clinician base, we calculated the number of physicians and advanced practitioners who prescribed buprenorphine per 1000 prescribers of any medication in each month. The denominator was restricted to Medicaid prescribers with at least 50 prescriptions over the study period to exclude clinicians who minimally participate in Medicaid. We assessed trends for prescribers overall and separately for physicians in primary care (PCPs), psychiatry, emergency medicine (EM), addiction medicine, and all other specialties as well as for advanced practitioners with any specialty. To examine whether the initiatives were associated with changes in the composition of prescribing clinician types, we calculated the monthly percentage of buprenorphine prescribers who were advanced practitioners.

### Statistical Analysis

Using aggregated monthly measures of buprenorphine receipt and prescribing in the New Jersey Medicaid population from 2017 to 2020, interrupted time series analyses compared outcomes before and after the initiatives went into effect in April 2019. Analyses were conducted using R statistical software, version 4.2.2 (R Project for Statistical Computing). We used the auto.arima function from the R package forecast^26^ to choose the best-fitting model, allowing for autoregressive and moving average parameters to be selected based on fit statistics. Models included a constant, a slope term to account for secular trends, terms to estimate changes in level and slope for the outcome measures, control variables, and any autoregressive or moving average effects. Prescriber-level models additionally controlled for the month of September 2019, as NPI information was missing from most prescription claims during that month only. All analyses used a 2-tailed significance threshold of *P* < .05. We performed several sensitivity analyses to assess robustness of findings to outcome definitions, study periods, and OUD medications. We also conducted a comparative interrupted time series analysis of aggregate Medicaid prescribing data (the Centers for Medicare & Medicaid Services State Drug Utilization Data),^27^ available nationally for the same time period, to assess whether our results could be explained by concurrent, non–state-specific secular trends in prescribing (eMethods in Supplement 1).

## Results

### Buprenorphine Receipt

Of 1 204 162 New Jersey Medicaid beneficiaries who met enrollment criteria during the study period, 101 423 had an OUD diagnosis (mean [SD] age, 41.0 [11.6] years; 54 726 [54.0%] male; 30 071 [29.6%] Black, 10 143 [10.0%] Hispanic, and 51 238 [50.5%] White), of whom 20 090 filled at least 1 buprenorphine prescription. The rate of buprenorphine receipt among Medicaid beneficiaries with OUD increased from 96.7 to 165.8 per 1000 beneficiaries from January 2017 to December 2020, an increase of 71.5%.

**Figure 1.**
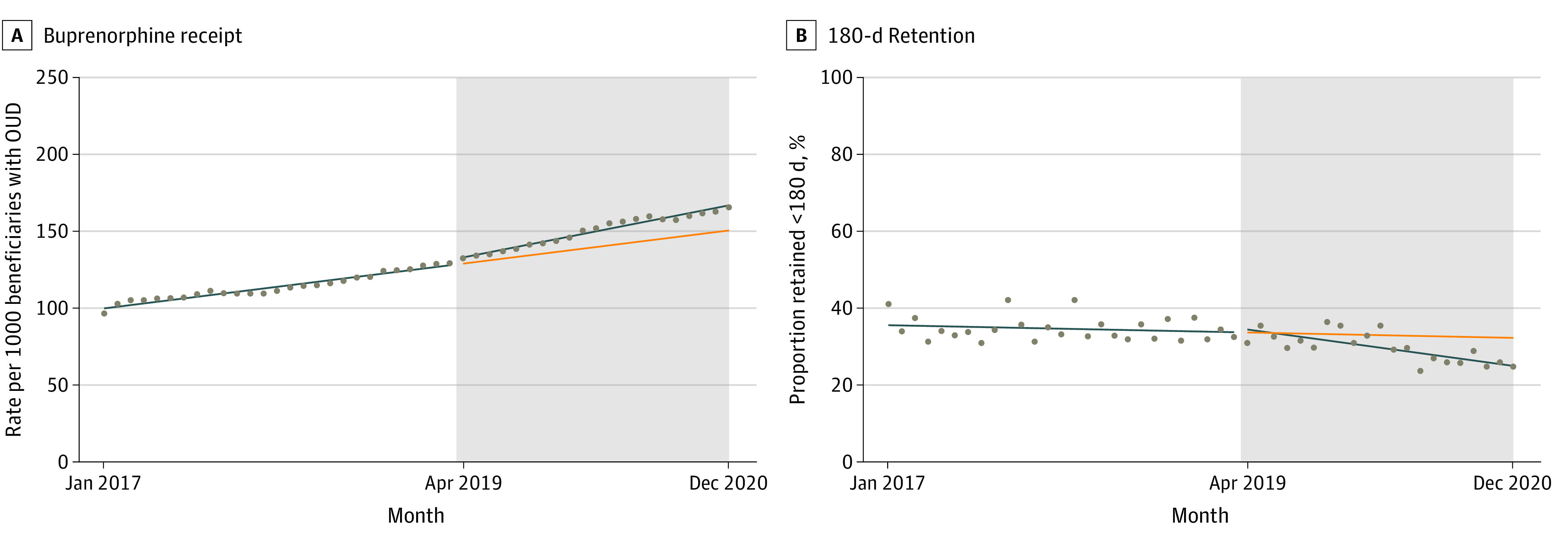
Trends in Buprenorphine Receipt and Retention Before and After Implementation of New Jersey Medicaid Initiatives Grey shaded areas represent postimplementation period. Blue line represents observed trends. Orange line represents the counterfactual (ie, projected postinitiative trends without implementation). Dots represent monthly observations.

Implementation was associated with a 36% increase in the monthly rate during the postpolicy period (0.47 per 1000 beneficiaries; 95% CI, 0.10-0.85 per 1000 beneficiaries), increasing the monthly trend to 1.76 (95% CI, 1.46-2.06) per 1000 beneficiaries. The initiatives were associated with no immediate change in buprenorphine receipt, but a significant increase in the monthly rate during the postpolicy period (0.47 per 1000 beneficiaries; 95% CI, 0.10-0.85 per 1000 beneficiaries) (Table 1 and Figure 1). In a sensitivity analysis of trends among the general Medicaid population, initiatives were associated with an immediate increase in buprenorphine receipt but no trend change (eTable 3 and eFigure 1 in Supplement 1). This difference likely reflects COVID-19–related changes in the Medicaid population^28^ resulting in a lower proportion of beneficiaries with OUD. Additional sensitivity analyses showed that trends in methadone, naltrexone, and any MOUD receipt associated with the initiatives differ from those of buprenorphine (eTable 4 and eFigure 2 in Supplement 1). A secondary analysis using national Medicaid Drug Utilization Data^27^ further showed that quarterly trends in buprenorphine prescriptions increased in New Jersey relative to combined data for all other states following initiative implementation (1.27 prescriptions per 1000 Medicaid beneficiaries; 95% CI, 0.58 to 1.96 per 1000 beneficiaries) (eTable 5 and eFigure 3 in Supplement 1).

**Table 1.** Interrupted Time Series Estimates for the Association of New Jersey Medicaid Initiatives With Buprenorphine Receipt and Retention

Outcome	Slope (95%CI)		
	Preinitiative trend	Changes associated with initiative	
		Immediate change	Trend change
Buprenorphine receipt, rate per 1000 beneficiaries with OUD	1.29 (1.02 to 1.56)	1.78 (−0.89 to 4.44)	0.47 (0.10 to 0.85)
New episodes with ≥180 d retention, %	0.02 (−0.19 to 0.24)	−0.08 (−4.12 to 3.96)	−0.34 (−0.75 to 0.07)

Abbreviation: OUD, opioid use disorder.

Among beneficiaries with new buprenorphine episodes, the trend in the percentage retained for 180 or more days was stable in the prepolicy period (0.02% per month; 95% CI, −0.19% to 0.24% per month). The initiatives were associated with no immediate change in retention, but we observed a decrease in the monthly trend in the percentage retained although it was not statistically significant (−0.34% per month; 95% CI, −0.75% to 0.07% per month) (Table 1 and Figure 1). In sensitivity analyses, the initiatives were not associated with an increase in the number of episodes lasting 180 or more days (eTable 3 and eFigure 1 in Supplement 1). Sensitivity analyses excluding the COVID-19 period and testing a COVID-19 effect suggest the trend decrease after implementation was associated with COVID-19 rather than the initiatives (eTable 6 and eFigure 4 in Supplement 1).

### Buprenorphine Prescribing

There were 1788 buprenorphine prescribers of 32 193 total physician or advanced practice prescribers with 50 or more prescriptions throughout the study period. The monthly rate of buprenorphine prescribing increased 63.2% during the study period, from 20.9 to 34.1 per 1000 prescribers.

**Figure 2.**
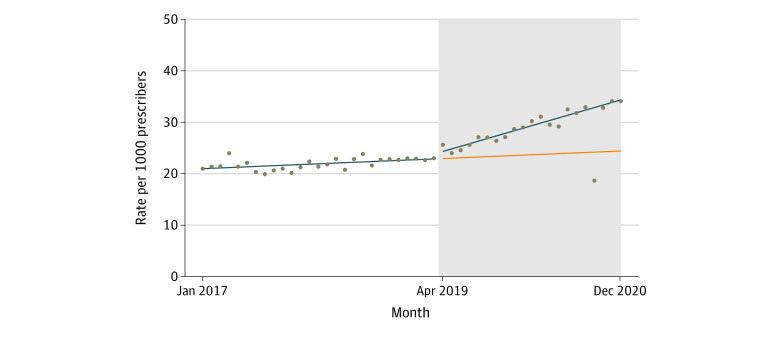
Trends in Buprenorphine Prescribing Rates per 1000 Total Medicaid Prescribers Before and After Implementation of NJ Medicaid Initiatives Grey shaded area represents postimplementation period. Blue line represents observed trends. Orange line represents the counterfactual (ie, projected postinitiative trends without implementation). Dots represent monthly observations.

The monthly number of overall buprenorphine prescribers was increasing in the prepolicy period (0.07 per 1000 prescribers; 95% CI, 0.03-0.12 per 1000 prescribers). The policy was associated with a monthly trend increase of 0.43 per 1000 prescribers (95% CI, 0.34 to 0.51 per 1000 prescribers) in the period after implementation relative to the preimplementation trend (Table 2 and Figure 2).

**Table 2.** Interrupted Time Series Estimates for the Association of New Jersey Medicaid Initiatives With Buprenorphine Prescribing Rates^a^

Prescriber type and specialty	Slope (95%CI)		
	Preinitiative trend	Changes associated with initiative	
		Immediate change	Trend change
All prescriber types	0.07 (0.03 to 0.12)	0.96 (−0.14 to 2.05)	0.43 (0.34 to 0.51)
Physicians: primary care	−0.04 (−0.10 to 0.01)	0.49 (−0.87 to 1.85)	0.42 (0.32 to 0.53)
Physicians: psychiatry	−0.10 (−0.37 to 0.18)	−1.29 (−7.84 to 5.27)	1.49 (1.00 to 1.99)
Physicians: emergency medicine	0.26 (0.09 to 0.43)	15.79 (11.69 to 19.90)	0.45 (0.14 to 0.76)
Advanced practitioners	0.60 (0.57 to 0.63)	−1.96 (−3.45 to −0.47)	0.42 (0.32 to 0.52)

^a^ Rates shown are per 1000 Medicaid prescribers in each category.

**Figure 3.**
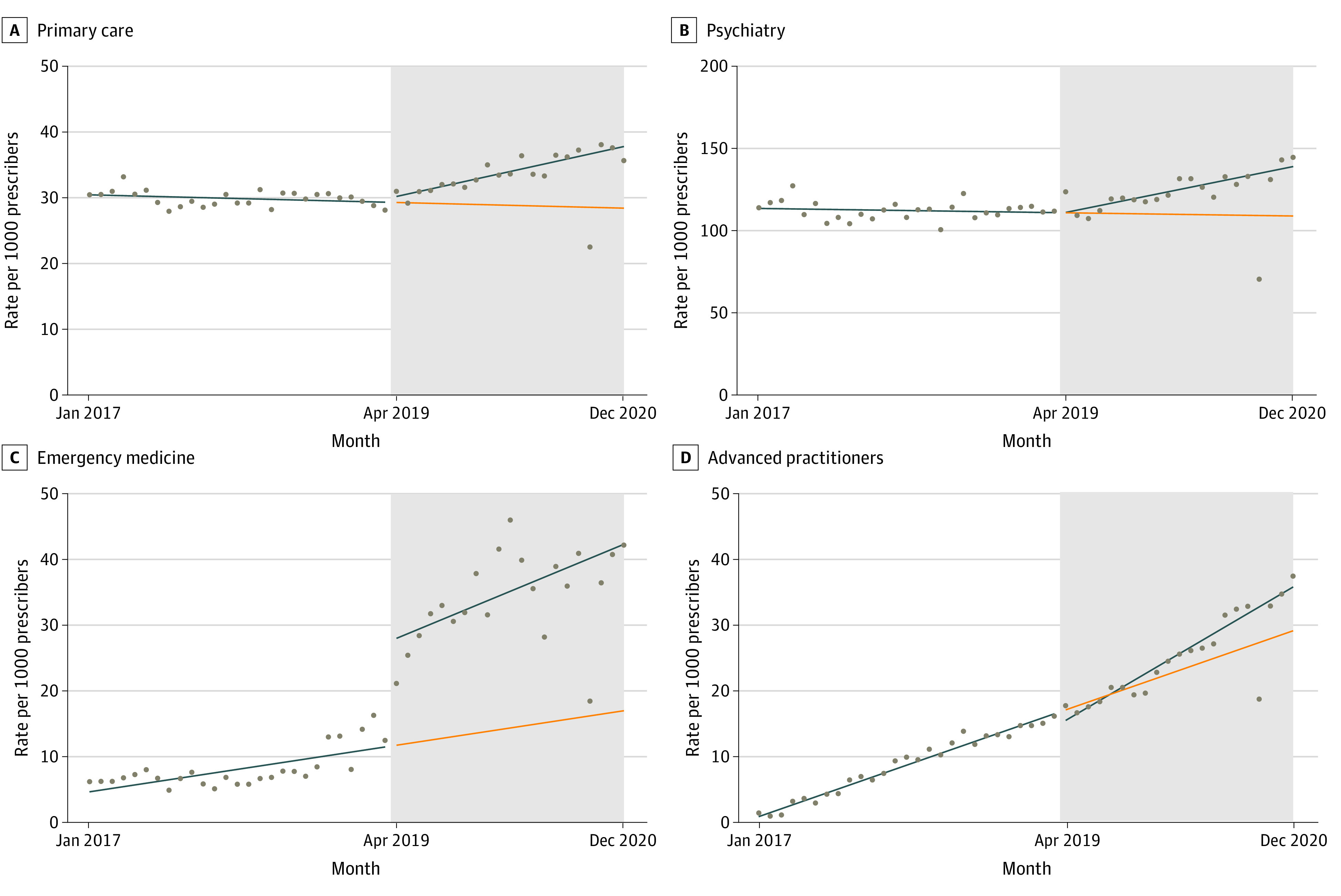
Trends in Buprenorphine Prescribing Rates per 1000 Total Medicaid Prescribers by Clinician Type and Specialty Before and After Implementation of New Jersey Medicaid Initiatives Grey shaded area represents postimplementation period. Blue line represents observed trends. Orange line represents the counterfactual (ie, projected postinitiative trends without implementation). Dots represent monthly observations.

In analyses stratified by prescriber type, initiatives were associated with significant increases in the monthly rate of buprenorphine prescribing for all types. Among PCPs, initiatives were associated with a trend increase of 0.42 per 1000 prescribers (95% CI, 0.32-0.53 per 1000 prescribers). Psychiatrists prescribed buprenorphine at higher rates at baseline and showed significant monthly rate increases following policy implementation (1.49 per 1000 prescribers; 95% CI, 1.00-1.99 per 1000 prescribers). EM physicians prescribed at low rates prior to policy implementation, but initiatives were associated with large immediate (15.79 per 1000 prescribers; 95% CI, 11.69-19.90 per 1000 prescribers) and trend (0.45 per 1000 prescribers; 0.14-0.76 per 1000 prescribers) increases. Buprenorphine prescribing among advanced practitioners increased at a monthly rate of 0.60 per 1000 prescribers (95% CI, 0.57-0.63 per 1000 prescribers) before policy implementation, and this trend accelerated after implementation (0.42 per 1000 prescribers; 95% CI, 0.32-0.52 per 1000 prescribers) (Table 2, Figure 3). Initiatives were not associated with increasing trends in buprenorphine prescribing among addiction medicine physicians (eTable 7 and eFigure 5 in Supplement 1) but were associated with a trend change among physicians with all other specialties (0.12 per 1000 prescribers; 95% CI, 0.07-0.16 per 1000 prescribers) (eTable 8 and eFigure 6 in Supplement 1). Findings were similar in sensitivity analyses modeling trends in prescriber counts, excluding observations from January to March 2019 and defining the outcome as the rate of prescribers per 1000 beneficiaries with OUD (eTables 9-11 and eFigures 7-10 in Supplement 1). There was no association of the initiatives with naltrexone prescribing (eTable 12 and eFigure 11 in Supplement 1).

Before policy implementation, advanced practitioners accounted for a growing percentage of buprenorphine prescribers, increasing by 0.58% per month (95% CI, 0.56% to 0.60%) from January 2017 to March 2019. Policy implementation was associated with a small immediate decline in this percentage (−1.70%; 95% CI, −2.56% to −0.84%), and a modest trend change (−0.06%; 95% CI, −0.12% to 0.00%) (eTable 13 and eFigure 12 in Supplement 1).

## Discussion

This study found that New Jersey Medicaid initiatives were associated with a trend shift in the proportion of beneficiaries with OUD receiving buprenorphine. Although the initiatives were not associated with an immediate increase at implementation, the growth rate over time in buprenorphine receipt increased by 36% in the period after implementation. The initiatives were also associated with increases in the number of buprenorphine prescribers, overall and within clinician types. Increases were most pronounced among PCPs and EM physicians. These results suggest that the initiatives may have reduced barriers to buprenorphine prescribing for clinicians and expanded access to treatment for patients with OUD.

Our findings add to growing evidence indicating that initiatives like those implemented in New Jersey can improve access to evidence-based care for people with OUD. Prior authorizations are a commonly cited barrier to buprenorphine prescribing,^29,30^ and eliminating prior authorization requirements has been shown to increase treatment access.^31,32^ Efforts like New Jersey’s OBAT program, which increase reimbursement and assist prescribers in addressing patients’ psychosocial needs, have similarly found success in expanding availability and utilization of treatment services.^33,34,35^ Support for buprenorphine prescribers like that provided by New Jersey’s 2 MAT COEs, can also increase buprenorphine prescribing.^35,36,37^ Importantly, synergistic factors unique to New Jersey may account for the changes in buprenorphine use and prescribing observed in this study. Similar opioid-related policies may have different outcomes across states, as policy effects depend on contextual factors and state-specific efforts.^38,39^ This supports the value of state-level studies that assess treatment trends following policy changes in individual states in which the overall policy context and concurrent developments are best understood and that avoids the difficulty in identifying relevant single-state comparators. The likely success of New Jersey’s initiatives may also reflect an environment that is generally supportive of MOUDs, as demonstrated by the state’s many efforts to increase MOUD access.

Buprenorphine prescribing trend increases were especially pronounced among PCPs and EM physicians. The increase among PCPs is consistent with policy goals, which targeted clinicians working in primary care and other general health care settings. Although none of the initiatives directly targeted EM physicians, the sharp increase observed for EM physicians could be explained by an emphasis on ED-based buprenorphine prescribing by the COEs and by large hospital systems, which deployed hundreds of peer navigators in EDs during the study period.^21,22^ While the primary focus of these efforts was to implement successful referral to community-based MOUDs, the increased focus on intervening with individuals experiencing overdose may also have encouraged emergency department (ED)–based buprenorphine prescribing to expedite treatment initiation and provide at least short-term protection from repeat overdose. Additionally, increasing buprenorphine availability in the community could mitigate the commonly reported barrier to ED-initiated buprenorphine of limited referral options.^40,41^ Other efforts to improve ED OUD care occurring during the same period may also have contributed to the increase in prescribing among EM physicians.^42^ Nonetheless, fewer than 50 of every 1000 EM physicians prescribed buprenorphine by the end of the study period, indicating substantial room for improvement given that those with OUD frequently present to EDs.^43,44^

Our results highlight the increasingly important role of advanced practitioners in prescribing buprenorphine. With the passage of the Comprehensive Addiction and Recovery Act in 2016 and then the SUPPORT Act in 2018,^45^ advanced practitioners became eligible to prescribe buprenorphine. Our analyses show that the percentage of advanced practitioners among all buprenorphine prescribers increased steadily, reaching 26% of Medicaid buprenorphine prescribers by December 2020. Other studies similarly demonstrate the important role of advanced practitioners in expanding access to buprenorphine.^5,46^ However, many states have scope-of-practice laws restricting prescribing by advanced practitioners, both buprenorphine-specific and general,^47^ which limit prescribing capacity.^46,48^ Greater regulatory flexibility for high-priority conditions like OUD could increase access to treatment.

We found that even as buprenorphine receipt increased, the percentage of new buprenorphine episodes lasting at least 180 days did not. Several explanations may account for this finding. First, initiatives were implemented less than a year before the COVID-19 pandemic began, which led to significant treatment disruptions despite rapidly implemented flexibilities to sustain care.^49^ Sensitivity analyses suggest that declines in retention after initiatives were implemented were likely attributed to COVID-19 rather than policy effects. Second, recent efforts to expand buprenorphine access may have changed the clinician landscape, with expansion to new settings including those that are fully virtual.^50^ Research is needed to evaluate whether treatment retention and other indicators of care quality vary across clinician types and settings. Third, characteristics of patients prescribed buprenorphine that affect treatment success may be changing with the evolving drug supply and as buprenorphine becomes more widely available. For example, greater availability may have increased the number of first-time treatment seekers, who have less experience navigating the treatment system and higher risk of early discontinuation. It remains critical to engage patients in care, even for shorter episodes, as longer buprenorphine retention is consistently associated with improved outcomes,^51,52^ overdose risk is substantially lowered during treatment periods,^53^ and buprenorphine episode duration increases with subsequent attempts.^54^ Additional research is needed to identify successful retention strategies, and payer and policy efforts are needed to support their uptake. Given the strong protective effect of MOUDs on overdose and other outcomes,^53,55,56^ future research should also examine whether initiatives like those implemented in New Jersey not only increase access to treatment, but reduce opioid-related consequences.

### Limitations

This study has limitations. The Medicaid initiatives examined in this state-level study were implemented concurrently, so it is not possible to determine separate policy effects. The lack of a direct comparison group is an additional limitation. However, inclusion of multiple outcomes with consistent results, clear trend shifts at the time of implementation, and consistent findings in sensitivity analyses, including an analysis comparing New Jersey buprenorphine prescribing trends with all other states, increase confidence in our results. The study period intersected with the COVID-19 pandemic, which may have affected trends. Analyses did not account for unmeasured patient characteristics that may affect outcomes, such as treatment readiness. Data are from a single state and payer and findings may not generalize to other contexts. In patient-level analyses, we required continuous Medicaid enrollment for 13 or more months; however, churn is common^57^ and findings may not represent those with coverage gaps.

## Conclusions

In this cross-sectional study of state-level New Jersey Medicaid initiatives designed to expand access to buprenorphine, policy initiatives were associated with increases in buprenorphine prescribing by clinicians and to patients with OUD. Across medical specialties and clinician types, the number of buprenorphine prescribers and beneficiaries with OUD receiving buprenorphine increased at a faster rate following policy implementation. However, the percentage of new buprenorphine episodes reaching 180 or more days did not significantly change, indicating that retention remains a challenge. Our findings support initiatives like those implemented in New Jersey but emphasize the need for efforts to support long-term retention.

## Notes

Supplement 1.
eMethods.
**eTable 1.** NDC and service codes used to identify medications for opioid use disorder **eTable 2.** Prescriber categories and taxonomy codes **eTable 3.** Interrupted time series estimates for the association of NJ Medicaid initiatives with buprenorphine receipt and retention **eTable 4.** Interrupted time series estimates for the association of NJ Medicaid initiatives with naltrexone, methadone, and any MOUD receipt **eTable 5.** Changes in Medicaid buprenorphine prescriptions before and after NJ Medicaid initiatives in NJ relative to all other US states **eTable 6.** Interrupted time series estimates for trends in buprenorphine retention associated with NJ Medicaid initiatives and COVID-19 **eTable 7.** Interrupted time series estimates for the association of NJ Medicaid initiatives with buprenorphine prescribing rates for addiction medicine physicians **eTable 8.** Interrupted time series estimates for the association of NJ Medicaid initiatives with buprenorphine prescribing rates for other physician specialties **eTable 9.** Interrupted time series estimates for the association of NJ Medicaid initiatives with number of buprenorphine prescribers **eTable 10.** Interrupted time series estimates for the association of NJ Medicaid initiatives with buprenorphine prescribers, with washout period from January to March 2019 **eTable 11.** Interrupted time series estimates for the association of NJ Medicaid initiatives with buprenorphine prescribers per 1000 Medicaid beneficiaries with OUD **eTable 12.** Interrupted time series estimates for the association of NJ Medicaid initiatives with naltrexone prescribing rates **eTable 13.** Interrupted time series estimates for the association of NJ Medicaid initiatives with percentage of advanced practitioner buprenorphine prescribers **eFigure 1.** Trends in buprenorphine receipt per 100 000 Medicaid beneficiaries (A) and number of patients retained 180+ days (B) before and after implementation of NJ Medicaid initiatives **eFigure 2.** Trends in naltrexone (A), methadone (B), and any MOUD (C) receipt before and after implementation of NJ Medicaid initiatives **eFigure 3.** Trends in Medicaid buprenorphine prescriptions before and after implementation of NJ Medicaid initiatives in NJ and all other US states **eFigure 4.** Trends in buprenorphine receipt before and after implementation of NJ Medicaid initiatives, excluding COVID-19 period (A) and trends in buprenorphine receipt before and during COVID-19 (B) **eFigure 5.** Trends in buprenorphine prescribing trends for addiction medicine physicians before and after implementation of NJ Medicaid initiatives, rate per 1000 prescribers (A) and number of prescribers (B) **eFigure 6.** Trends in buprenorphine prescribing trends for other physician specialties before and after implementation of NJ Medicaid initiatives, rate per 1000 prescribers (A) and number of prescribers (B) **eFigure 7.** Trends in the overall number of Medicaid buprenorphine prescribers before and after implementation of NJ Medicaid initiatives **eFigure 8.** Trends in the number of Medicaid buprenorphine prescribers by provider type and specialty before and after implementation of NJ Medicaid initiatives **eFigure 9.** Trends in buprenorphine prescribers before and after implementation of NJ Medicaid initiatives, with washout period from January to March 2019 **eFigure 10.** Trends in buprenorphine prescribers before and after implementation of NJ Medicaid initiatives, rate per 1000 Medicaid beneficiaries with OUD **eFigure 11.** Trends in naltrexone prescribing rates per 1000 total Medicaid prescribers before and after implementation of NJ Medicaid initiatives **eFigure 12.** Trends in percentage of advanced practitioner buprenorphine prescribers before and after implementation of NJ Medicaid initiatives
eReferences.

Supplement 2.
Data Sharing Statement

## References

[1] The White House. National Drug Control Strategy. Accessed March 20, 2023. https://www.whitehouse.gov/ondcp/national-drug-control-strategy

[2] National Academies of Sciences, Engineering, and Medicine. *Medications for Opioid Use Disorder Save Lives*. National Academies Press; 2019. Accessed March 28, 2023. https://nap.nationalacademies.org/catalog/25310/medications-for-opioid-use-disorder-save-lives 30896911

[3] Mauro PM, Gutkind S, Annunziato EM, Samples H. Use of medication for opioid use disorder among us adolescents and adults with need for opioid treatment, 2019. JAMA Netw Open. 2022;5(3):e223821. doi:10.1001/jamanetworkopen.2022.3821 35319762 PMC8943638

[4] Donohue JM, Jarlenski MP, Kim JY, ; Medicaid Outcomes Distributed Research Network (MODRN). Use of medications for treatment of opioid use disorder among US Medicaid enrollees in 11 states, 2014-2018. JAMA. 2021;326(2):154-164. doi:10.1001/jama.2021.7374 34255008 PMC8278273

[5] Saunders H, Britton E, Cunningham P, . Medicaid participation among practitioners authorized to prescribe buprenorphine. J Subst Abuse Treat. 2022;133:108513. doi:10.1016/j.jsat.2021.108513 34148758

[6] Roehler DR, Guy GP Jr, Jones CM. Buprenorphine prescription dispensing rates and characteristics following federal changes in prescribing policy, 2017-2018: A cross-sectional study. Drug Alcohol Depend. 2020;213:108083. doi:10.1016/j.drugalcdep.2020.108083 32505044 PMC9590643

[7] Stein BD, Saloner B, Schuler MS, Gurvey J, Sorbero M, Gordon AJ. Concentration of patient care among buprenorphine-prescribing clinicians in the US. JAMA. 2021;325(21):2206-2208. doi:10.1001/jama.2021.4469 34061152 PMC8170540

[8] Olfson M, Zhang V, Schoenbaum M, King M. Buprenorphine treatment by primary care providers, psychiatrists, addiction specialists, and others. Health Aff (Millwood). 2020;39(6):984-992. doi:10.1377/hlthaff.2019.01622 32479224 PMC9097821

[9] Abraham R, Wilkinson E, Jabbarpour Y, Petterson S, Bazemore A. Characteristics of office-based buprenorphine prescribers for Medicare patients. J Am Board Fam Med. 2020;33(1):9-16. doi:10.3122/jabfm.2020.01.190233 31907241

[10] Mackey K, Veazie S, Anderson J, Bourne D, Peterson K. Barriers and facilitators to the use of medications for opioid use disorder: a rapid review. J Gen Intern Med. 2020;35(3)(suppl 3):954-963. doi:10.1007/s11606-020-06257-4 33145687 PMC7728943

[11] Winograd RP, Coffey B, Woolfolk C, . To prescribe or not to prescribe? barriers and motivators for progressing along each stage of the buprenorphine training and prescribing path. J Behav Health Serv Res. 2023;50(2):165-180. doi:10.1007/s11414-021-09783-z 35060002

[12] Foti K, Heyward J, Tajanlangit M, . Primary care physicians’ preparedness to treat opioid use disorder in the United States: A cross-sectional survey. Drug Alcohol Depend. 2021;225:108811. doi:10.1016/j.drugalcdep.2021.108811 34175786 PMC10659122

[13] Fiscella K, Wakeman SE, Beletsky L. Buprenorphine deregulation and mainstreaming treatment for opioid use disorder: X the X waiver. JAMA Psychiatry. 2019;76(3):229-230. doi:10.1001/jamapsychiatry.2018.3685 30586140

[14] Lanham HJ, Papac J, Olmos DI, . Survey of barriers and facilitators to prescribing buprenorphine and clinician perceptions on the Drug Addiction Treatment Act of 2000 waiver. JAMA Netw Open. 2022;5(5):e2212419. doi:10.1001/jamanetworkopen.2022.12419 35552721 PMC9099423

[15] Decker SL. In 2011 nearly one-third of physicians said they would not accept new Medicaid patients, but rising fees may help. Health Aff (Millwood). 2012;31(8):1673-1679. doi:10.1377/hlthaff.2012.0294 22869644 PMC6292513

[16] Wen H, Wilk AS, Druss BG, Cummings JR. Medicaid acceptance by psychiatrists before and after Medicaid expansion. JAMA Psychiatry. 2019;76(9):981-983. doi:10.1001/jamapsychiatry.2019.0958 31166578 PMC6551583

[17] Orgera K, Tolbert J. The opioid epidemic and Medicaid’s role in facilitating access to treatment. Kaiser Family Foundation. May 24, 2019. Accessed March 20, 2023. https://www.kff.org/medicaid/issue-brief/the-opioid-epidemic-and-medicaids-role-in-facilitating-access-to-treatment

[18] State of New Jersey, Department of Human Services, Division of Medical Assistance & Health Services. Newsletter: office based addictions treatment (OBAT) and elimination of prior authorization for medication assisted treatment (MAT) for all MAT providers. November 2019. Accessed March 20, 2023. https://www.njmmis.com/downloadDocuments/29-18.pdf

[19] Abraham AJ, Andrews CM, Harris SJ, Westlake MM, Grogan CM. Coverage and prior authorization policies for medications for opioid use disorder in Medicaid managed care. JAMA Health Forum. 2022;3(11):e224001. doi:10.1001/jamahealthforum.2022.4001 36331441 PMC10157383

[20] State of New Jersey, Department of Human Services, Division of Medical Assistance & Health Services. Newsletter: Office Based Addiction Treatment (OBAT) update—enrollment of OBAT navigators as servicing providers. March 2020. Accessed March 20, 2023. https://www.njmmis.com/downloadDocuments/30-03.pdf

[21] Northern New Jersey MAT Center of Excellence. Accessed March 20, 2023. https://sites.rutgers.edu/mat-coe

[22] Southern New Jersey MAT Center of Excellence. Accessed March 20, 2023. https://www.snjmatcoe.org

[23] Tunney S; NJ Department of Human Services, Division of Medical Assistance and Health Services. NJ MATrx Model. January 16, 2019. Accessed March 20, 2023. https://www.nj.gov/humanservices/dmhas/information/provider/Provider_Meetings/2019/MAAC%20OBAT.pdf

[24] US Centers for Medicare & Medicaid Services. NPPES NPI Registry. Accessed March 20, 2023. https://npiregistry.cms.hhs.gov

[25] National Uniform Claim Committee. Health care provider taxonomy. Accessed March 20, 2023. https://www.nucc.org/index.php/code-sets-mainmenu-41/provider-taxonomy-mainmenu-40

[26] Hyndman R. Package ‘forecast.’ February 27, 2023. Accessed March 20, 2023. https://cran.r-project.org/web/packages/forecast/forecast.pdf

[27] Centers for Medicare & Medicaid Services. State drug utilization data. December 19, 2022. Accessed March 20, 2023. https://www.medicaid.gov/medicaid/prescription-drugs/state-drug-utilization-data/index.html

[28] Khorrami P, Sommers BD. Changes in US Medicaid enrollment during the COVID-19 pandemic. JAMA Netw Open. 2021;4(5):e219463. doi:10.1001/jamanetworkopen.2021.9463 33950210 PMC8100862

[29] Marino LA, Campbell AN, Nunes EV, Sederer LI, Dixon LB. Factors influencing buprenorphine prescribing among physicians in New York State. J Addict. 2019;2019:7832752. doi:10.1155/2019/7832752 31934492 PMC6942852

[30] Andraka-Christou B, Capone MJ. A qualitative study comparing physician-reported barriers to treating addiction using buprenorphine and extended-release naltrexone in U.S. office-based practices. Int J Drug Policy. 2018;54:9-17. doi:10.1016/j.drugpo.2017.11.021 29324253

[31] Mark TL, Parish WJ, Zarkin GA. Association of formulary prior authorization policies with buprenorphine-naloxone prescriptions and hospital and emergency department use among Medicare beneficiaries. JAMA Netw Open. 2020;3(4):e203132. doi:10.1001/jamanetworkopen.2020.3132 32310285 PMC7171554

[32] Andrews CM, Abraham AJ, Grogan CM, Westlake MA, Pollack HA, Friedmann PD. Impact of Medicaid restrictions on availability of buprenorphine in addiction treatment programs. Am J Public Health. 2019;109(3):434-436. doi:10.2105/AJPH.2018.304856 30676789 PMC6366513

[33] Cunningham P, Pierre-Louis S, Britton E, Farzana Urmi A, Barnes A. Addiction and Recovery Treatment Services: evaluation report for state fiscal years 2019 and 2020. Virginia Commonwealth University, School of Medicine. 2022. Accessed March 28, 2023. https://hbp.vcu.edu/media/hbp/policybriefs/pdfs/ARTSYear4ComprehensiveReport.5.4.22.pdf

[34] Harrison JM, Kerber R, Andraka-Christou B, Sorbero M, Stein BD. State policies and buprenorphine prescribing by nurse practitioners and physician assistants. Med Care Res Rev. 2022;79(6):789-797. doi:10.1177/10775587221086489 35435071 PMC10088360

[35] Kaplan-Dobbs M, Kattan JA, Tuazon E, Jimenez C, Saleh S, Kunins HV. Increasing access to buprenorphine in safety-net primary care clinics: the New York City Buprenorphine Nurse Care Manager Initiative. Am J Public Health. 2021;111(2):215-218. doi:10.2105/AJPH.2020.306000 33351661 PMC7811081

[36] Anderson JB, Martin SA, Gadomski A, . Project ECHO and primary care buprenorphine treatment for opioid use disorder: implementation and clinical outcomes. Subst Abus. 2022;43(1):222-230. doi:10.1080/08897077.2021.1931633 34086529

[37] Puckett HM, Bossaller JS, Sheets LR. The impact of project ECHO on physician preparedness to treat opioid use disorder: a systematic review. Addict Sci Clin Pract. 2021;16(1):6. doi:10.1186/s13722-021-00215-z 33482906 PMC7821394

[38] Keshwani S, Maguire M, Goodin A, Lo-Ciganic WH, Wilson DL, Hincapie-Castillo JM. Buprenorphine use trends following removal of prior authorization policies for the treatment of opioid use disorder in 2 state Medicaid programs. JAMA Health Forum. 2022;3(6):e221757. doi:10.1001/jamahealthforum.2022.1757 35977240 PMC9233239

[39] Allen L, Burns M, Saloner B. The consequences of removing prior authorization for buprenorphine in Medicaid—building an evidence base. JAMA Health Forum. 2022;3(6):e220189. doi:10.1001/jamahealthforum.2022.0189 36219020

[40] Lowenstein M, Kilaru A, Perrone J, . Barriers and facilitators for emergency department initiation of buprenorphine: a physician survey. Am J Emerg Med. 2019;37(9):1787-1790. doi:10.1016/j.ajem.2019.02.025 30803850 PMC7556325

[41] Zuckerman M, Kelly T, Heard K, Zosel A, Marlin M, Hoppe J. Physician attitudes on buprenorphine induction in the emergency department: results from a multistate survey. Clin Toxicol (Phila). 2021;59(4):279-285. doi:10.1080/15563650.2020.1805461 32870039

[42] Samuels EA, D’Onofrio G, Huntley K, . A quality framework for emergency department treatment of opioid use disorder. Ann Emerg Med. 2019;73(3):237-247. doi:10.1016/j.annemergmed.2018.08.439 30318376 PMC6817947

[43] Zhang X, Wang N, Hou F, . Emergency department visits by patients with substance use disorder in the United States. West J Emerg Med. 2021;22(5):1076-1085. doi:10.5811/westjem.2021.3.50839 34546883 PMC8463055

[44] Duber HC, Barata IA, Cioè-Peña E, . Identification, management, and transition of care for patients with opioid use disorder in the emergency department. Ann Emerg Med. 2018;72(4):420-431. doi:10.1016/j.annemergmed.2018.04.007 29880438 PMC6613583

[45] SUPPORT for Patients and Communities Act, HR 6, 115th Congress (2017-2018). Accessed March 20, 2023. https://www.congress.gov/bill/115th-congress/house-bill/6

[46] Barnett ML, Lee D, Frank RG. In rural areas, buprenorphine waiver adoption since 2017 driven by nurse practitioners and physician assistants. Health Aff (Millwood). 2019;38(12):2048-2056. doi:10.1377/hlthaff.2019.00859 31794302 PMC6938159

[47] Andraka-Christou B, Gordon AJ, Spetz J, . Beyond state scope of practice laws for advanced practitioners: Additional supervision requirements for buprenorphine prescribing. J Subst Abuse Treat. 2022;138:108715. doi:10.1016/j.jsat.2021.108715 35067400 PMC9167216

[48] Nguyen T, Muench U, Andraka-Christou B, Simon K, Bradford WD, Spetz J. The association between scope of practice regulations and nurse practitioner prescribing of buprenorphine after the 2016 Opioid Bill. Med Care Res Rev. 2022;79(2):290-298. doi:10.1177/10775587211004311 33792414 PMC8594929

[49] Treitler PC, Bowden CF, Lloyd J, Enich M, Nyaku AN, Crystal S. Perspectives of opioid use disorder treatment providers during COVID-19: adapting to flexibilities and sustaining reforms. J Subst Abuse Treat. 2022;132:108514. doi:10.1016/j.jsat.2021.108514 34098210 PMC8630075

[50] Butcher L. Does online opioid treatment work? Knowable Mag. Published online May 2, 2022. doi:10.1146/knowable-042922-1

[51] Williams AR, Samples H, Crystal S, Olfson M. Acute care, prescription opioid use, and overdose following discontinuation of long-term buprenorphine treatment for opioid use disorder. Am J Psychiatry. 2020;177(2):117-124. doi:10.1176/appi.ajp.2019.19060612 31786933 PMC7002204

[52] Samples H, Williams AR, Crystal S, Olfson M. Impact of long-term buprenorphine treatment on adverse health care outcomes in Medicaid. Health Aff (Millwood). 2020;39(5):747-755. doi:10.1377/hlthaff.2019.01085 32364847 PMC7531057

[53] Crystal S, Nowels M, Samples H, Olfson M, Williams AR, Treitler P. Opioid overdose survivors: Medications for opioid use disorder and risk of repeat overdose in Medicaid patients. Drug Alcohol Depend. 2022;232:109269. doi:10.1016/j.drugalcdep.2022.109269 35038609 PMC8943804

[54] Banta-Green CJ, Hansen RN, Ossiander EM, Wasserman CR, Merrill JO. Buprenorphine utilization among all Washington State residents’ based upon prescription monitoring program data—characteristics associated with two measures of retention and patterns of care over time. J Subst Abuse Treat. 2021;127:108446. doi:10.1016/j.jsat.2021.108446 34049724

[55] Santo T Jr, Clark B, Hickman M, . Association of opioid agonist treatment with all-cause mortality and specific causes of death among people with opioid dependence: a systematic review and meta-analysis. JAMA Psychiatry. 2021;78(9):979-993. doi:10.1001/jamapsychiatry.2021.0976 34076676 PMC8173472

[56] Sordo L, Barrio G, Bravo MJ, . Mortality risk during and after opioid substitution treatment: systematic review and meta-analysis of cohort studies. BMJ. 2017;357:j1550. doi:10.1136/bmj.j1550 28446428 PMC5421454

[57] Sugar S, Peters C, De Lew N, Sommers BD. Medicaid churning and continuity of care: evidence and policy considerations before and after the COVID-19 pandemic (Issue Brief No. HP-2021-10). Office of the Assistant Secretary for Planning and Evaluation, US Department of Health and Human Services. April 21, 2021. Accessed March 20, 2023. https://aspe.hhs.gov/sites/default/files/private/pdf/265366/medicaid-churning-ib.pdf

