# Supplementary material for: Buprenorphine Utilization and Prescribing Among New Jersey Medicaid Beneficiaries After Adoption of Initiatives Designed to Improve Treatment Access
Source: JAMA Netw Open. 2023 May 5;6(5):e2312030. doi: 10.1001/jamanetworkopen.2023.12030 (PMC10163388; doi:10.1001/jamanetworkopen.2023.12030)
Supplement: Supplement 1. — eMethods. eTable 1. NDC and service codes used to identify medications for opioid use disorder eTable 2. Prescriber categories and taxonomy codes eTable 3. Interrupted time series estimates for the association of NJ Medicaid initiatives with buprenorphine receipt and retention eTable 4. Interrupted time series estimates for the association of NJ Medicaid initiatives with naltrexone, methadone, and any MOUD receipt eTable 5. Changes in Medicaid buprenorphine prescriptions before and after NJ Medicaid initiatives in NJ relative to all other US states eTable 6. Interrupted time series estimates for trends in buprenorphine retention associated with NJ Medicaid initiatives and COVID-19 eTable 7. Interrupted time series estimates for the association of NJ Medicaid initiatives with buprenorphine prescribing rates for addiction medicine physicians eTable 8. Interrupted time series estimates for the association of NJ Medicaid initiatives with buprenorphine prescribing rates for other physician specialties eTable 9. Interrupted time series estimates for the association of NJ Medicaid initiatives with number of buprenorphine prescribers eTable 10. Interrupted time series estimates for the association of NJ Medicaid initiatives with buprenorphine prescribers, with washout period from January to March 2019 eTable 11. Interrupted time series estimates for the association of NJ Medicaid initiatives with buprenorphine prescribers per 1000 Medicaid beneficiaries with OUD eTable 12. Interrupted time series estimates for the association of NJ Medicaid initiatives with naltrexone prescribing rates eTable 13. Interrupted time series estimates for the association of NJ Medicaid initiatives with percentage of advanced practitioner buprenorphine prescribers eFigure 1. Trends in buprenorphine receipt per 100 000 Medicaid beneficiaries (A) and number of patients retained 180+ days (B) before and after implementation of NJ Medicaid initiatives eFigure 2. Trends in naltrexone (A), methadone (B [file jamanetwopen-e2312030-s001.pdf]

# Supplemental Online Content

Treitler P, Nowels M, Samples H, Crystal S. Buprenorphine utilization and prescribing among New Jersey Medicaid beneficiaries after adoption of initiatives designed to improve treatment access. *JAMA Netw Open.* 2023;6(5):e2312030. doi:10.1001/jamanetworkopen.2023.12030

**eMethods.**

**eTable 1.** NDC and service codes used to identify medications for opioid use disorder

**eTable 2.** Prescriber categories and taxonomy codes

**eTable 3.** Interrupted time series estimates for the association of NJ Medicaid initiatives with buprenorphine receipt and retention

**eTable 4.** Interrupted time series estimates for the association of NJ Medicaid initiatives with naltrexone, methadone, and any MOUD receipt

**eTable 5.** Changes in Medicaid buprenorphine prescriptions before and after NJ Medicaid initiatives in NJ relative to all other US states

**eTable 6.** Interrupted time series estimates for trends in buprenorphine retention associated with NJ Medicaid initiatives and COVID-19

**eTable 7.** Interrupted time series estimates for the association of NJ Medicaid initiatives with buprenorphine prescribing rates for addiction medicine physicians

**eTable 8.** Interrupted time series estimates for the association of NJ Medicaid initiatives with buprenorphine prescribing rates for other physician specialties

**eTable 9.** Interrupted time series estimates for the association of NJ Medicaid initiatives with number of buprenorphine prescribers

**eTable 10.** Interrupted time series estimates for the association of NJ Medicaid initiatives with buprenorphine prescribers, with washout period from January to March 2019

**eTable 11.** Interrupted time series estimates for the association of NJ Medicaid initiatives with buprenorphine prescribers per 1000 Medicaid beneficiaries with OUD

**eTable 12.** Interrupted time series estimates for the association of NJ Medicaid initiatives with naltrexone prescribing rates

**eTable 13.** Interrupted time series estimates for the association of NJ Medicaid initiatives with percentage of advanced practitioner buprenorphine prescribers

**eFigure 1.** Trends in buprenorphine receipt per 100 000 Medicaid beneficiaries (A) and number of patients retained 180+ days (B) before and after implementation of NJ Medicaid initiatives

**eFigure 2.** Trends in naltrexone (A), methadone (B), and any MOUD (C) receipt before and after implementation of NJ Medicaid initiatives

**eFigure 3.** Trends in Medicaid buprenorphine prescriptions before and after implementation of NJ Medicaid initiatives in NJ and all other US states

© 2023 Treitler P et al. *JAMA Network Open.*

**eFigure 4.** Trends in buprenorphine receipt before and after implementation of NJ Medicaid initiatives, excluding COVID-19 period (A) and trends in buprenorphine receipt before and during COVID-19 (B)

**eFigure 5.** Trends in buprenorphine prescribing trends for addiction medicine physicians before and after implementation of NJ Medicaid initiatives, rate per 1000 prescribers (A) and number of prescribers (B)

**eFigure 6.** Trends in buprenorphine prescribing trends for other physician specialties before and after implementation of NJ Medicaid initiatives, rate per 1000 prescribers (A) and number of prescribers (B)

**eFigure 7.** Trends in the overall number of Medicaid buprenorphine prescribers before and after implementation of NJ Medicaid initiatives

**eFigure 8.** Trends in the number of Medicaid buprenorphine prescribers by provider type and specialty before and after implementation of NJ Medicaid initiatives

**eFigure 9.** Trends in buprenorphine prescribers before and after implementation of NJ Medicaid initiatives, with washout period from January to March 2019

**eFigure 10.** Trends in buprenorphine prescribers before and after implementation of NJ Medicaid initiatives, rate per 1000 Medicaid beneficiaries with OUD

**eFigure 11.** Trends in naltrexone prescribing rates per 1000 total Medicaid prescribers before and after implementation of NJ Medicaid initiatives

**eFigure 12.** Trends in percentage of advanced practitioner buprenorphine prescribers before and after implementation of NJ Medicaid initiatives

**eReferences.**

This supplemental material has been provided by the authors to give readers additional information about their work.

© 2023 Treitler P et al. *JAMA Network Open.*

## eMethods

### Description of Sensitivity Analyses

We performed a range of sensitivity analyses to assess robustness to outcome definitions, study periods, OUD medications, and to examine whether trends in NJ buprenorphine prescription trends differed from those of other U.S. states during the study period. All model specifications (e.g., selection criteria, study variables) are as described in the manuscript Methods section unless otherwise noted below. With the exception of the analysis comparing buprenorphine prescriptions in NJ to all other U.S. states, all other sensitivity analyses used the NJ Medicaid dataset described in the manuscript.

***Sensitivity analyses for buprenorphine receipt and retention***

1. Association of NJ Medicaid initiatives with buprenorphine receipt unconditional on having an OUD diagnosis in the current or previous 12 months, based on prior research showing that a substantial proportion of patients receive treatment without a documented diagnosis.[1,2] Uses a denominator of all Medicaid beneficiaries meeting study enrollment criteria, with the exception of the requirement for OUD diagnosis in the current or prior 12 months (eTable 3, eFigure 1).
2. Association of NJ Medicaid initiatives with 180-day buprenorphine retention, where the outcome is modeled as the number of new episodes reaching 180+ days (eTable 3, eFigure 1).
3. Association of NJ Medicaid initiatives with receipt of naltrexone, methadone, and any MOUD (eTable 4, eFigure 2). Uses the same denominator (i.e., Medicaid enrollees with OUD meeting study criteria) as in primary analyses. Receipt of MOUD determined using NDC and service codes in eTable 1.
4. Association of NJ Medicaid initiatives with 180-day buprenorphine retention, excluding the COVID-19 period from March-December 2020 (eTable 6, eFigure 4).
5. Association of COVID-19 public health emergency declared in March 2020 with 180-day buprenorphine retention (eTable 6, eFigure 4).

***Sensitivity analysis comparing buprenorphine prescriptions in NJ to all other U.S. states***

We performed a comparative interrupted time series analysis[3] to determine whether buprenorphine prescription trends before and after the NJ initiatives differed between NJ and all other U.S. states. We used 2017-2020 buprenorphine prescription data from the Centers for Medicare and Medicaid Services (CMS) State Drug Utilization Data files,[4] which contains the number of prescriptions issued for each National Drug Code (NDC) on a state-quarter basis. We included only NDCs for buprenorphine formulations primarily indicated for OUD (eTable 1). For each quarter, we calculated the total number of prescriptions for the quarter, excluding cells with fewer than 11, which are suppressed by CMS and were set to 0 for this analysis. To account for changes in the Medicaid population over time, we calculated buprenorphine prescription rates as the number of prescriptions per 1,000 Medicaid beneficiaries in each state-quarter. The number of beneficiaries was calculated using monthly Medicaid enrollment counts from the Kaiser Family Foundation’s Total Monthly Medicaid/CHIP Enrollment database,[5] by averaging counts from the three months that comprised each quarter.

Next, we fit a segmented regression model as specified in Lopez Bernal et al.[3] The key effects of interest are 1) the relative pre-policy trend, which is the difference in trend in the prescription rate between NJ and all other states; 2) the relative intercept change, or the level shift in prescription rate occurring at the time of implementation for NJ relative to all other states; and 3) the trend change, or the change in time trend from before to after implementation for NJ relative to all other states. Results of this analysis show that buprenorphine prescription trends in NJ were similar to those in the main analysis, with an increasing trend before the initiatives went into effect and a further increase in the magnitude of the trend after initiatives went into effect. In comparison states, buprenorphine prescriptions also increased during the pre-initiative period, but the trend flattened in the post-policy period. Results of this analysis are shown in eTable 5 and eFigure 3.

© 2023 Treitler P et al. *JAMA Network Open.*

***Sensitivity analyses for buprenorphine prescribing:***

1. Association of NJ Medicaid initiatives with the number of buprenorphine prescribers, overall and by provider type (eTable 9, eFigures 7-8).
2. Association of NJ Medicaid initiatives with buprenorphine prescribers per 1,000 total prescribers, in which January – March 2019 was considered a transition period and excluded from analyses, with pre-policy trends calculated using data through December 2018 (eTable 10, eFigure 9).
3. Association of NJ Medicaid initiatives with buprenorphine prescribers per 1,000 Medicaid beneficiaries with OUD (using the same denominator as in patient-level analyses of buprenorphine receipt) (eTable 11, eFigure 10).
4. Association of NJ Medicaid initiatives with naltrexone prescribers, shown as a rate per 1,000 total Medicaid prescribers in each category (eTable 12, eFigure 11).

© 2023 Treitler P et al. *JAMA Network Open.*

## eTable 1. NDC and service codes used to identify medications for opioid use disorder

| |
|---|
| **Buprenorphine prescription NDC codes:**<br>00378876516, 00378876593, 00378876616, 00378876693, 00378876716, 00378876793, 00378876816, 00378876893, 00781721606, 00781721664, 00781722706, 00781722764, 00781723806, 00781723864, 00781724906, 00781724964, 12496120201, 12496120203, 12496120401, 12496120403, 12496120801, 12496120803, 12496121201, 12496121203, 43598057901, 43598057930, 43598058001, 43598058030, 43598058101, 43598058130, 43598058201, 43598058230, 47781035503, 47781035511, 47781035603, 47781035611, 47781035703, 47781035711, 47781035803, 47781035811, 52427069203, 52427069211, 52427069403, 52427069411, 52427069803, 52427069811, 52427071203, 52427071211, 54569639900, 55700014730, 59385001201, 59385001230, 59385001401, 59385001430, 59385001601, 59385001630, 52440010014, 58284010014, 12496010001, 12496010002, 12496010005, 12496030001, 12496030002, 12496030005, 00054017613, 00054017713, 00054018813, 00054018913, 00093537856, 00093537956, 00093572056, 00093572156, 00228315303, 00228315403, 00228315473, 00228315503, 00228315567, 00228315573, 00228315603, 00378092393, 00378092493, 00406192303, 00406192403, 00406800503, 00406802003, 12496127802, 12496128302, 12496130602, 12496131002, 16590066630, 35356000407, 35356000430, 35356055530, 35356055630, 42291017430, 42291017530, 42858050103, 42858050203, 43063018407, 43063018430, 43063066706, 43063075306, 49999039507, 49999039515, 49999039530, 49999063830, 49999063930, 50268014411, 50268014415, 50268014511, 50268014515, 50383028793, 50383029493, 50383092493, 50383093093, 52959030430, 52959074930, 54123011430, 54123090730, 54123091430, 54123092930, 54123095730, 54123098630, 54569549600, 54569573900, 54569573901, 54569573902, 54569640800, 54868570700, 54868570701, 54868570702, 54868570703, 54868570704, 54868575000, 55045378403, 55700018430, 55700030230, 55700030330, 60429058611, 60429058630, 60429058633, 60429058711, 60429058730, 60429058733, 60687048111, 60687048121, 60687049211, 60687049221, 62175045232, 62175045832, 62756045983, 62756046083, 62756096983, 62756097083, 63629402801, 63629403401, 63629403402, 63629403403, 63874108403, 63874108503, 63874117303, 65162041503, 65162041603, 66336001630, 68071138003, 68071151003, 68258299903, 68308020230, 68308020830, 71335115403 |
| **Naltrexone prescription NDC codes:**<br>00056001122, 00056001130, 00056001170, 00056007950, 00056008050, 00185003901, 00185003930, 00406009201, 00406009203, 00406117001, 00406117003, 00555090201, 00555090202, 00904703604, 16729008101, 16729008110, 42291063230, 43063059115, 47335032683, 47335032688, 50090286600, 50436010501, 51224020630, 51224020650, 51285027501, 51285027502, 52152010502, 52152010504, 52152010530, 54868557400, 63459030042, 63629104601, 63629104701, 65694010003, 65694010010, 65757030001, 65757030202, 68084029111, 68084029121, 68094085362, 68115068030<br>**Naltrexone HCPCS service code:** J2315 |
| **Methadone HCPCS service codes:**<br>H0020, Z2006, Z3357, H0018HFU1, H0019HFU1 |

© 2023 Treitler P et al. *JAMA Network Open.*

## eTable 2. Prescriber categories and taxonomy codes

| Category | Taxonomy codes | Taxonomy description |
|---|---|---|
| Physicians: primary care | 207Q00000X | Family Medicine |
| | 207QA0000X | Family Medicine: Adolescent Medicine |
| | 207QA0505X | Family Medicine: Adult Medicine |
| | 207QG0300X | Family Medicine: Geriatric Medicine |
| | 208D00000X | General Practice |
| | 207R00000X | Internal Medicine |
| | 207RA0000X | Internal Medicine: Adolescent Medicine |
| | 207RG0300X | Internal Medicine: Geriatric Medicine |
| | 208000000X | Pediatrics |
| | 2080A0000X | Pediatrics: adolescent medicine |
| Physicians: psychiatry | 2084B0040X | Psychiatry & Neurology: Behavioral Neurology & Neuropsychiatry |
| | 2084P0804X | Psychiatry & Neurology: Child & Adolescent Psychiatry & Neurology: Psychiatry |
| | 2084F0202X | Psychiatry & Neurology: Forensic Psychiatry |
| | 2084P0805X | Psychiatry & Neurology: Geriatric Psychiatry |
| | 2084B0002X | Psychiatry & Neurology: Obesity Medicine |
| | 2084P0800X | Psychiatry & Neurology: Psychiatry |
| | 2084P0015X | Psychiatry & Neurology: Psychosomatic Medicine |
| Physicians: emergency medicine | 207P00000X<br>207PE0004X<br>207PH0002X<br>207PT0002X<br>207PP0204X<br>207PS0010X<br>207PE0005X | All taxonomy codes with Grouping of "Allopathic & Osteopathic Physicians" and Classification of "Emergency Medicine." |
| Physicians: addiction medicine | 207LA0401X | Anesthesiology: Addiction Medicine |
| | 207QA0401X | Family Medicine: Addiction Medicine |
| | 207RA0401X | Internal Medicine: Addiction Medicine |
| | 2083A0300X | Preventive Medicine: Addiction Medicine |
| | 2084A0401X | Psychiatry and Neurology: Addiction Medicine |
| | 2084P0802X | Psychiatry and Neurology: Addiction Psychiatry |
| Physicians: other specialties | All other physician taxonomy codes | All taxonomy codes with Grouping of "Allopathic & Osteopathic Physicians" not included in above categories. |
| Advanced practitioners | All advanced practitioner taxonomy codes | All taxonomy codes with Grouping of "Physician Assistants & Advanced Practice Nursing Providers." |

Taxonomy codes are from the National Uniform Claim Committee, Health Care Provider Taxonomy Code Set Version 23.0, 1/1/23. https://nucc.org/index.php/code-sets-mainmenu-41/provider-taxonomy-mainmenu-40/csv-mainmenu-57

**eTable 3. Interrupted time series estimates for the association of NJ Medicaid initiatives with buprenorphine receipt and retention**

| | | Changes associated with policy | |
|---|---|---|---|
| | Pre-policy trend | Immediate change | Trend change |
| **Outcome** | **Slope (95% CI)** | **Slope (95% CI)** | **Slope (95% CI)** |
| Buprenorphine receipt, rate per 100,000 Medicaid beneficiaries | 12.13 (9.54 to 14.71) | 25.75 (3.30 to 48.19) | 1.76 (-3.62 to 7.14) |
| New episodes lasting 180+ days, N | 2.31 (1.27 to 3.36) | 9.54 (-8.01 to 27.10) | -1.45 (-3.19 to 0.28) |

© 2023 Treitler P et al. *JAMA Network Open.*

**eTable 4. Interrupted time series estimates for the association of NJ Medicaid initiatives with naltrexone, methadone, and any MOUD receipt**

| | | Changes associated with event | |
|---|---|---|---|
| | Pre-policy trend | Immediate change | Trend change |
| **Outcome** | **Slope (95% CI)** | **Slope (95% CI)** | **Slope (95% CI)** |
| Naltrexone receipt, rate per 1,000 beneficiaries with OUD | 0.11 (-0.01 to 0.22) | 1.74 (0.53 to 2.96) | -0.34 (-0.49 to -0.19) |
| Methadone receipt, rate per 1,000 beneficiaries with OUD | 1.06 (0.74 to 1.38) | 4.76 (2.09 to 7.44) | -0.49 (-1.00 to 0.03) |
| Any MOUD receipt, rate per 1,000 beneficiaries with OUD[a] | 2.48 (2.07 to 2.90) | 8.62 (5.14 to 12.09) | -0.38 (-1.05 to 0.28) |

[a] Any MOUD includes buprenorphine, naltrexone, and methadone.

© 2023 Treitler P et al. *JAMA Network Open.*

## eTable 5. Changes in Medicaid buprenorphine prescriptions before and after NJ Medicaid initiatives in NJ relative to all other US states[a]

| | Trends in NJ relative to all other U.S. states | | |
|---|---|---|---|
| **Outcome** | **Relative pre-policy trend (95% CI)[b]** | **Relative intercept change (95% CI)[c]** | **Relative trend change (95% CI)[d]** |
| Buprenorphine prescriptions per 1,000 Medicaid beneficiaries | -1.69 (-2.08, -1.30) | -3.16 (-6.30, -0.03) | 1.27 (0.58, 1.96) |

[a] Analysis uses data from the Centers for Medicare and Medicaid Services (CMS) State Drug Utilization Data files (see eMethods for details).
[b] Relative pre-policy trend is the difference in trend in the prescription rate between NJ and all other states
[c] Relative intercept change is the level shift in prescription rate occurring at the time of implementation for NJ relative to all other states.
[d] Trend change is the change in time trend from before to after implementation for NJ relative to all other states.

© 2023 Treitler P et al. *JAMA Network Open.*

## eTable 6. Interrupted time series estimates for trends in buprenorphine retention associated with NJ Medicaid initiatives and COVID-19

| | | Changes associated with event | |
|---|---|---|---|
| | Pre-policy trend | Immediate change | Trend change |
| **Outcome** | **Slope (95% CI)** | **Slope (95% CI)** | **Slope (95% CI)** |
| % of new episodes with 180+ day retention, excluding COVID-19 period[a] | 0.03 (-0.15 to 0.22) | -3.43 (-7.30 to 0.43) | 0.28 (-0.24 to 0.80) |
| % of new episodes with 180+ day retention, modeling COVID-19 effect[b] | 0.00 (-0.15 to 0.15) | -5.70 (-10.55 to -0.85) | -0.29 (-0.96 to 0.37) |

[a] Immediate and trend changes are relative to NJ Medicaid initiative implementation in April 2019. Excludes period after COVID-19 public health emergency declaration (March-December 2020).
[b] Immediate and trend changes are relative to COVID-19 public health emergency declaration in March 2020.

© 2023 Treitler P et al. *JAMA Network Open.*

**eTable 7. Interrupted time series estimates for the association of NJ Medicaid initiatives with buprenorphine prescribing rates for addiction medicine physicians**

| | Pre-policy trend | Changes associated with policy | |
|---|---|---|---|
| | | Immediate change | Trend change |
| Prescriber type and specialty | Slope (95% CI) | Slope (95% CI) | Slope (95% CI) |
| Addiction medicine physicians, rate per 1,000 prescribers | 1.23 (-0.83 to 3.29) | 4.36 (-46.83 to 55.55) | 0.67 (-3.13 to 4.47) |
| Addiction medicine physicians, number of prescribers | 0.49 (0.29 to 0.70) | 1.21 (-1.95 to 4.37) | -0.51 (-0.90 to -0.13) |

© 2023 Treitler P et al. *JAMA Network Open.*

**eTable 8. Interrupted time series estimates for the association of NJ Medicaid initiatives with buprenorphine prescribing rates for other physician specialties[a]**

| | Pre-policy trend | Changes associated with policy: Immediate change | Changes associated with policy: Trend change |
|---|---|---|---|
| Prescriber type and specialty | Slope (95% CI) | Slope (95% CI) | Slope (95% CI) |
| Other physician specialties, rate per 1,000 prescribers | -0.04 (-0.06, -0.01) | 1.20 (0.65 to 1.76) | 0.12 (0.07 to 0.16) |
| Other physician specialties, number of prescribers | 0.21 (0.03 to 0.40) | 8.05 (3.66 to 12.44) | 0.32 (-0.01 to 0.65) |

[a] Other physician specialties include all specialties other than primary care, psychiatry, emergency medicine, and addiction medicine. Grey shaded area represents post-implementation period.

© 2023 Treitler P et al. *JAMA Network Open.*

**eTable 9. Interrupted time series estimates for the association of NJ Medicaid initiatives with number of buprenorphine prescribers**

| | | Changes associated with policy | |
|---|---|---|---|
| | Pre-policy trend | Immediate change | Trend change |
| Prescriber type and specialty | Slope (95% CI) | Slope (95% CI) | Slope (95% CI) |
| All prescriber types | 5.15 (4.55 to 5.75) | 13.74 (-0.58 to 28.07) | 5.80 (4.71 to 6.89) |
| Physicians: primary care | 0.13 (-0.16 to 0.42) | 2.69 (-4.19 to 9.57) | 1.89 (1.37 to 2.41) |
| Physicians: psychiatry | 0.44 (0.20 to 0.69) | -5.95 (-11.79 to -0.11) | 1.31 (0.87 to 1.75) |
| Physicians: emergency medicine | 0.40 (0.13 to 0.66) | 18.90 (12.84 to 24.95) | 0.26 (-0.22 to 0.73) |
| Advanced practitioners | 3.23 (3.09 to 3.38) | -6.13 (-12.60 to 0.33) | 2.63 (2.19 to 3.08) |

© 2023 Treitler P et al. *JAMA Network Open.*

**eTable 10. Interrupted time series estimates for the association of NJ Medicaid initiatives with buprenorphine prescribers, with washout period from January to March 2019**

| | | Changes associated with policy | |
|---|---|---|---|
| | Pre-policy trend | Immediate change | Trend change |
| Outcome | Slope (95% CI) | Slope (95% CI) | Slope (95% CI) |
| Buprenorphine prescribers per 1,000 Medicaid prescribers | 0.07 (0.02 to 0.12) | 1.00 (-0.16 to 2.15) | 0.43 (0.34 to 0.51) |

© 2023 Treitler P et al. *JAMA Network Open.*

**eTable 11. Interrupted time series estimates for the association of NJ Medicaid initiatives with buprenorphine prescribers per 1000 Medicaid beneficiaries with OUD**

| | | Changes associated with policy | |
|---|---|---|---|
| | Pre-policy trend | Immediate change | Trend change |
| Outcome | Slope (95% CI) | Slope (95% CI) | Slope (95% CI) |
| Buprenorphine prescribers per 1,000 Medicaid beneficiaries with OUD | 0.06 (0.02 to 0.10) | 1.14 (0.46 to 1.81) | 0.09 (0.00 to 0.17) |

© 2023 Treitler P et al. *JAMA Network Open.*

**eTable 12. Interrupted time series estimates for the association of NJ Medicaid initiatives with naltrexone prescribing rates[a]**

| | | Changes associated with policy | |
|---|---|---|---|
| | Pre-policy trend | Immediate change | Trend change |
| Prescriber type and specialty | Slope (95% CI) | Slope (95% CI) | Slope (95% CI) |
| All prescriber types | 0.22 (0.16, 0.29) | -0.02 (-1.46, 1.41) | 0.01 (-0.11, 0.12) |
| Physicians: primary care | 0.31 (0.21, 0.40) | 0.29 (-1.63, 2.21) | -0.19 (-0.36, -0.02) |
| Physicians: psychiatry | 0.47 (0.05, 0.94) | 2.34 (-8.27, 12.95) | 0.07 (-0.78, 0.93) |
| Physicians: emergency medicine | 0.17 (0.14, 0.20) | -0.70 (-2.20, 0.81) | -0.04 (-0.15, 0.06) |
| Advanced practitioners | 0.37 (0.28, 0.45) | -1.31 (-3.31, 0.69) | 0.20 (0.05, 0.35) |

[a] Rates shown are per 1,000 Medicaid prescribers in each category.

**eTable 13. Interrupted time series estimates for the association of NJ Medicaid initiatives with percentage of advanced practitioner buprenorphine prescribers**

| | | Changes associated with policy | |
|---|---|---|---|
| | Pre-policy trend | Immediate change | Trend change |
| Outcome | Slope (95% CI) | Slope (95% CI) | Slope (95% CI) |
| Advanced practitioners, % of all prescribers | 0.58 (0.56 to 0.60) | -1.70 (-2.56 to -0.84) | -0.06 (-0.12 to 0.00) |

© 2023 Treitler P et al. *JAMA Network Open.*

**eFigure 1. Trends in buprenorphine receipt per 100 000 Medicaid beneficiaries (A) and number of patients retained 180+ days (B) before and after implementation of NJ Medicaid initiatives**

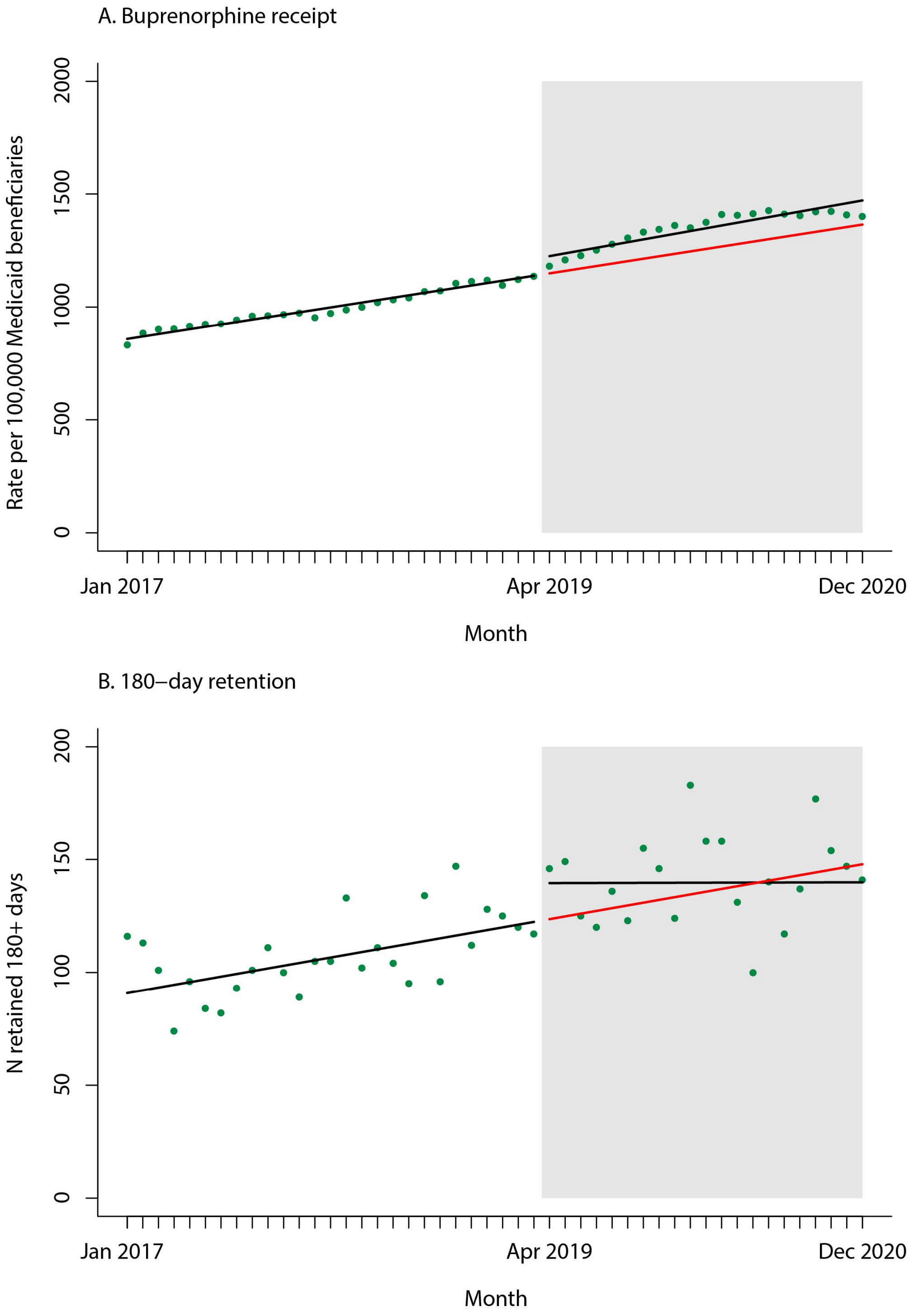


Grey shaded area represents post-implementation period. Black line represents observed trends. Red line represents the counterfactual (i.e., projected post-initiative trends without implementation).

© 2023 Treitler P et al. *JAMA Network Open.*

**eFigure 2. Trends in naltrexone (A), methadone (B), and any MOUD (C) receipt before and after implementation of NJ Medicaid initiatives**

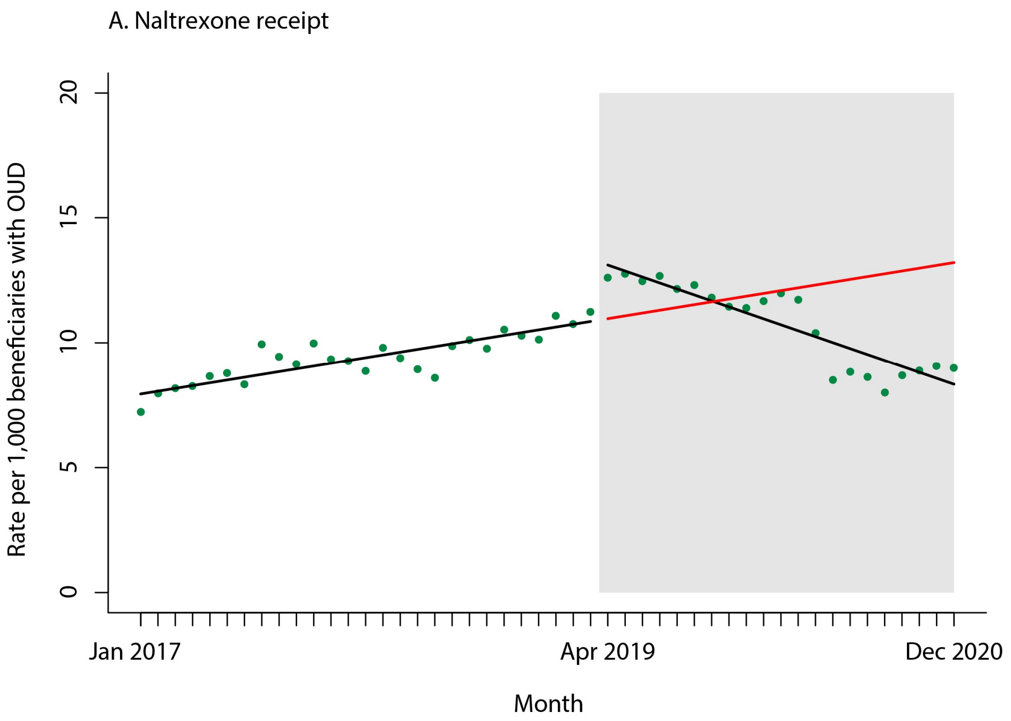


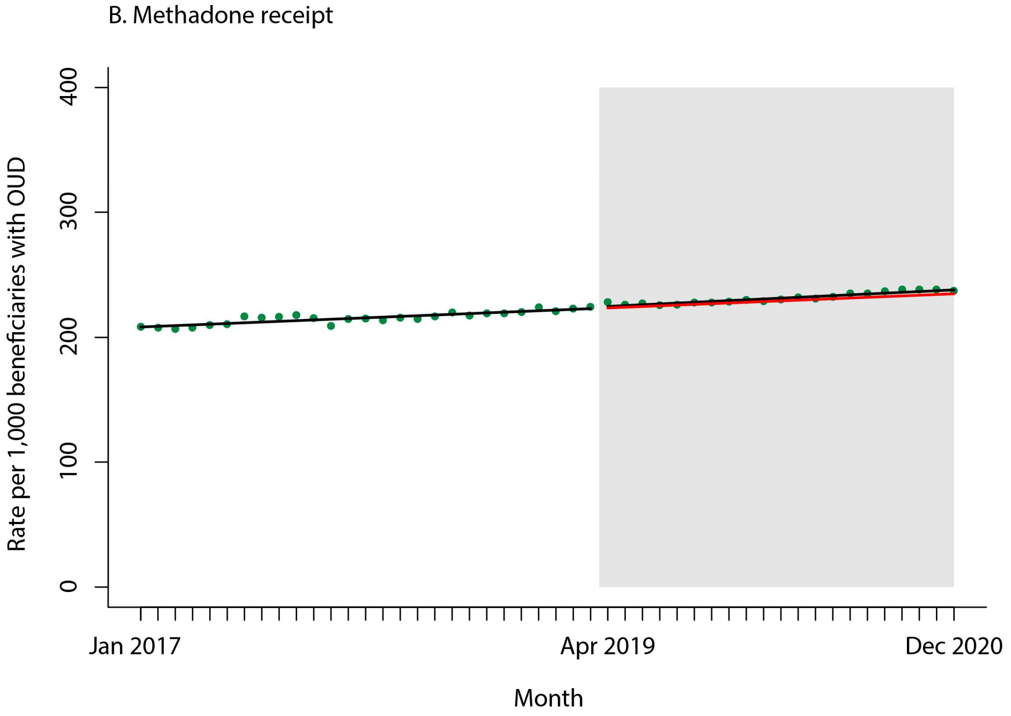


© 2023 Treitler P et al. *JAMA Network Open.*

C. Any MOUD receipt

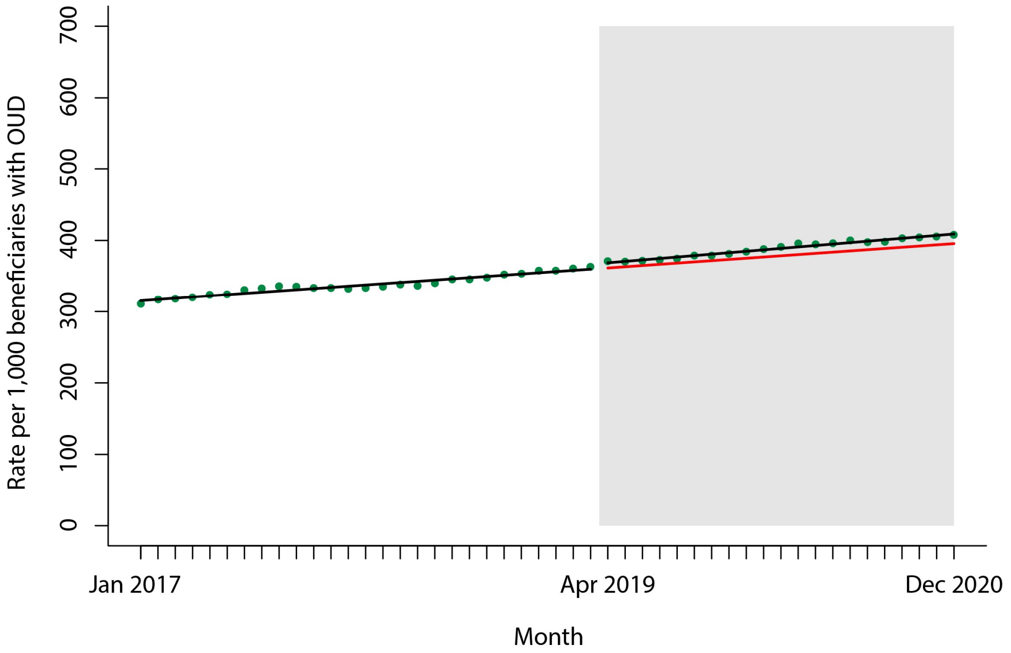


Grey shaded area represents post-implementation period. Black line represents observed trends. Red line represents the counterfactual (i.e., projected post-initiative trends without implementation).

© 2023 Treitler P et al. *JAMA Network Open.*

## eFigure 3. Trends in Medicaid buprenorphine prescriptions before and after implementation of NJ Medicaid initiatives in NJ and all other US states

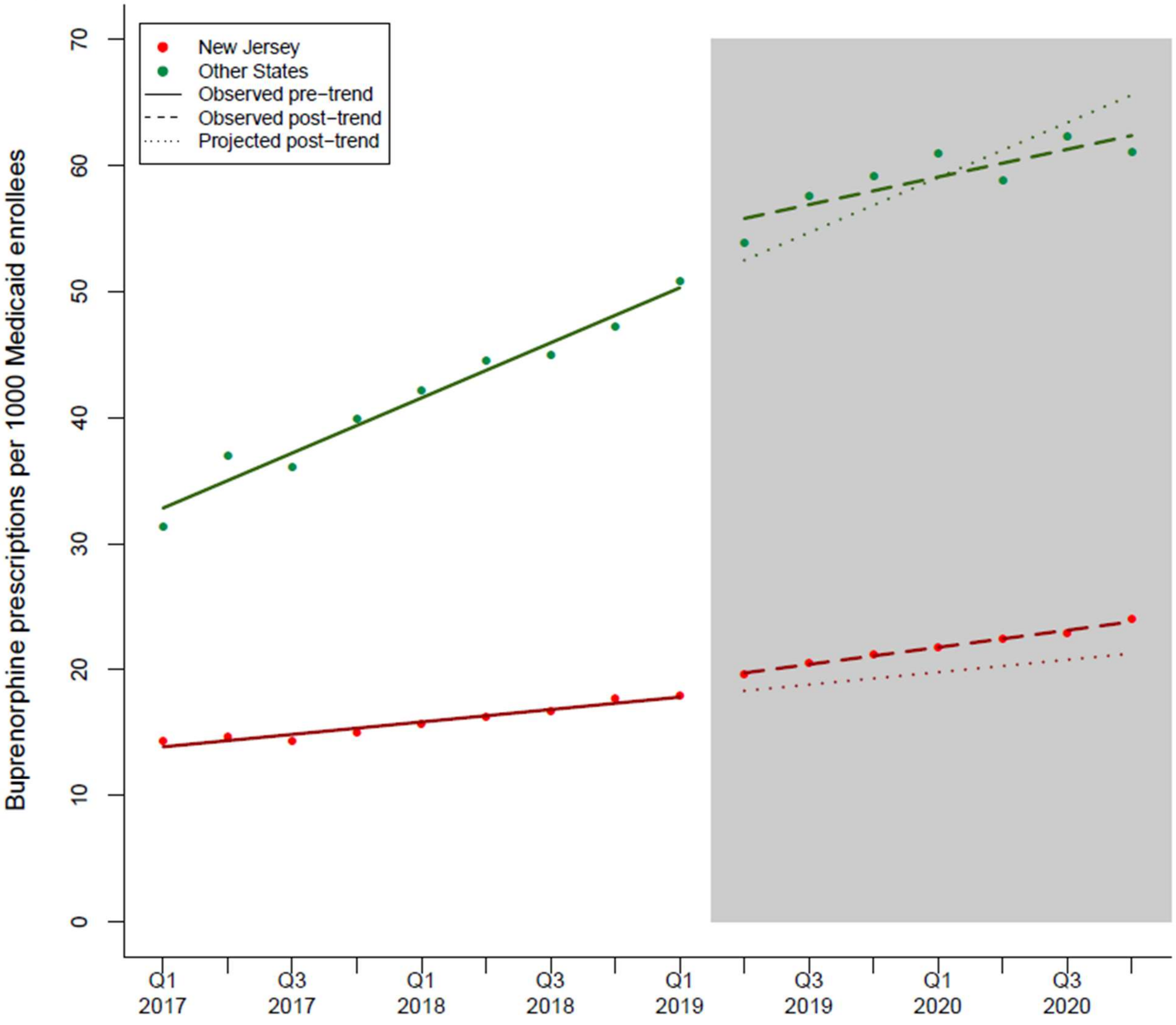


Analysis uses data from the Centers for Medicare and Medicaid Services (CMS) State Drug Utilization Data files (see eMethods for details). Grey shaded area represents post-implementation period. Black line represents observed trends. Red line represents the counterfactual (i.e., projected post-initiative trends without implementation). Estimates of relative differences in trends are shown in eTable 5.

© 2023 Treitler P et al. *JAMA Network Open.*

**eFigure 4. Trends in buprenorphine receipt before and after implementation of NJ Medicaid initiatives, excluding COVID-19 period (A) and trends in buprenorphine receipt before and during COVID-19 (B)**

A. Excluding COVID-19 period

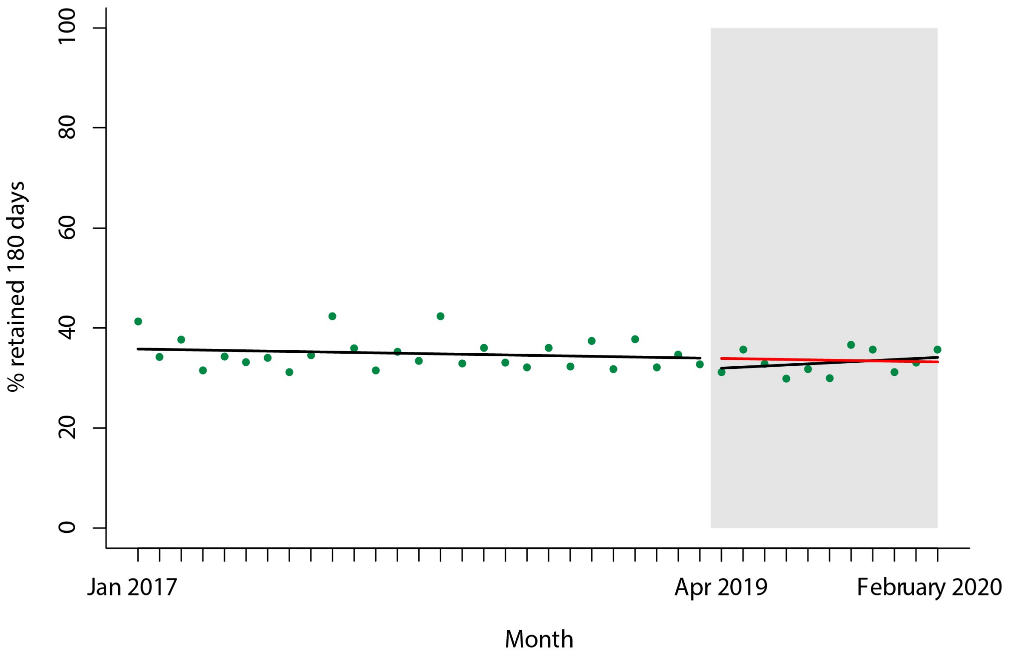


B. COVID-19 effect

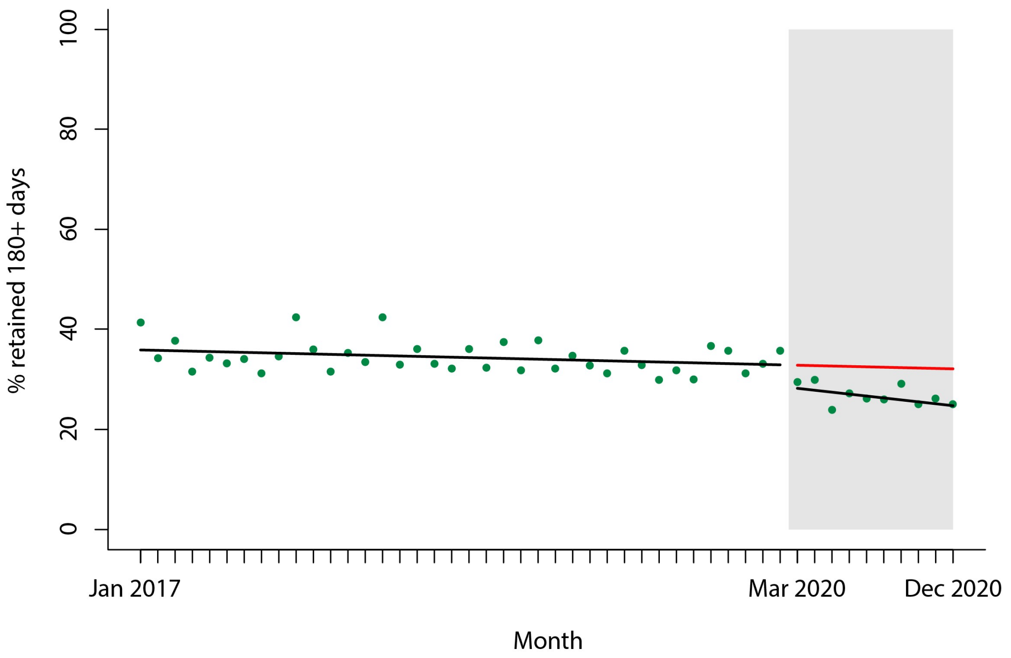


Grey shaded area represents post-implementation period. Black line represents observed trends. Red line represents the counterfactual (i.e., projected post-initiative trends without implementation).

© 2023 Treitler P et al. *JAMA Network Open.*

**eFigure 5. Trends in buprenorphine prescribing trends for addiction medicine physicians before and after implementation of NJ Medicaid initiatives, rate per 1000 prescribers (A) and number of prescribers (B)**

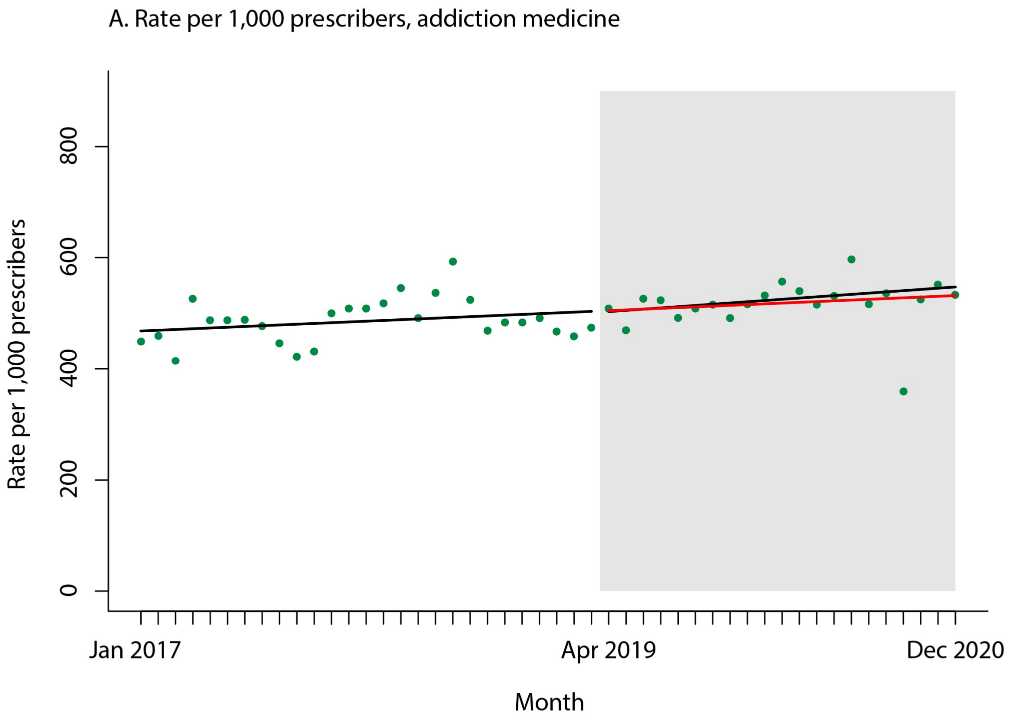


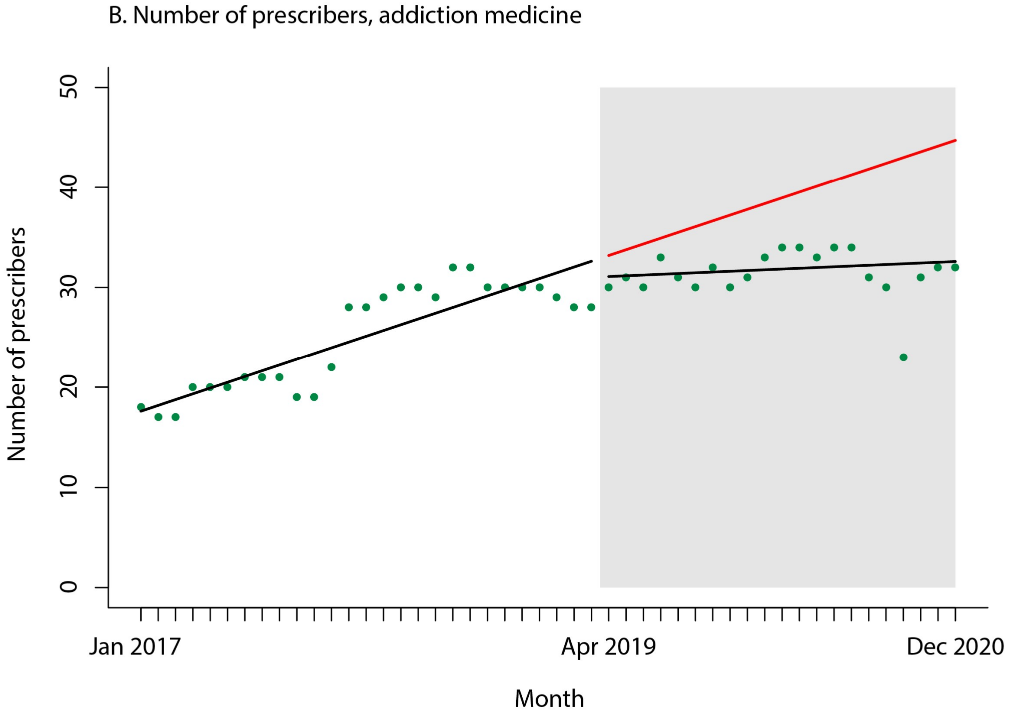


Grey shaded area represents post-implementation period. Black line represents observed trends. Red line represents the counterfactual (i.e., projected post-initiative trends without implementation).

© 2023 Treitler P et al. *JAMA Network Open.*

**eFigure 6. Trends in buprenorphine prescribing trends for other physician specialties before and after implementation of NJ Medicaid initiatives, rate per 1000 prescribers (A) and number of prescribers (B)**

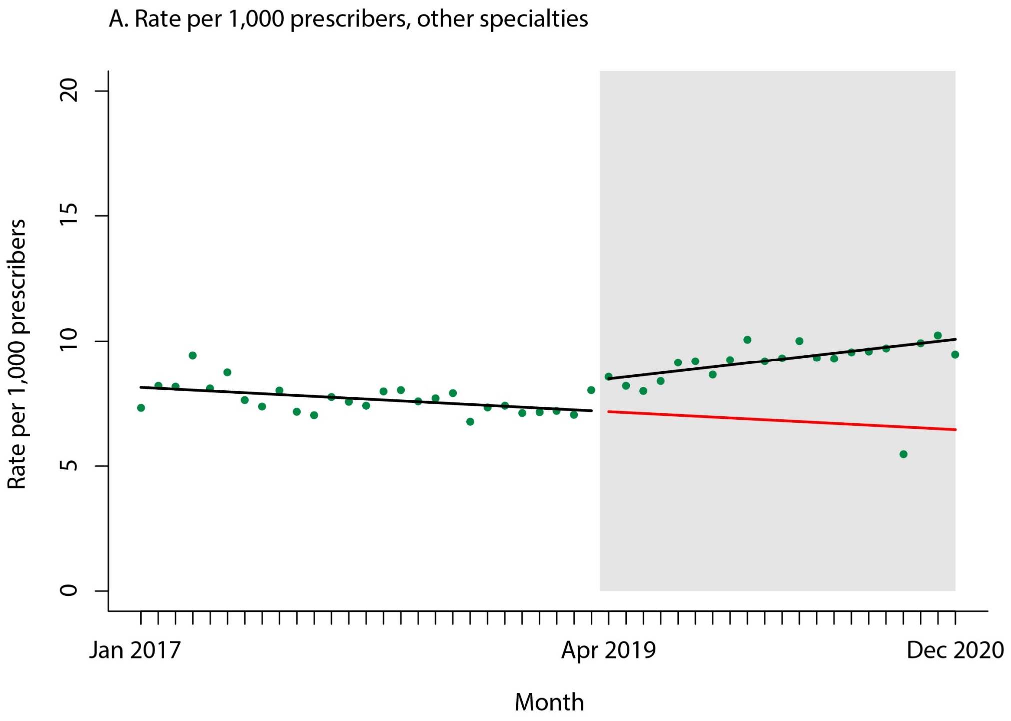


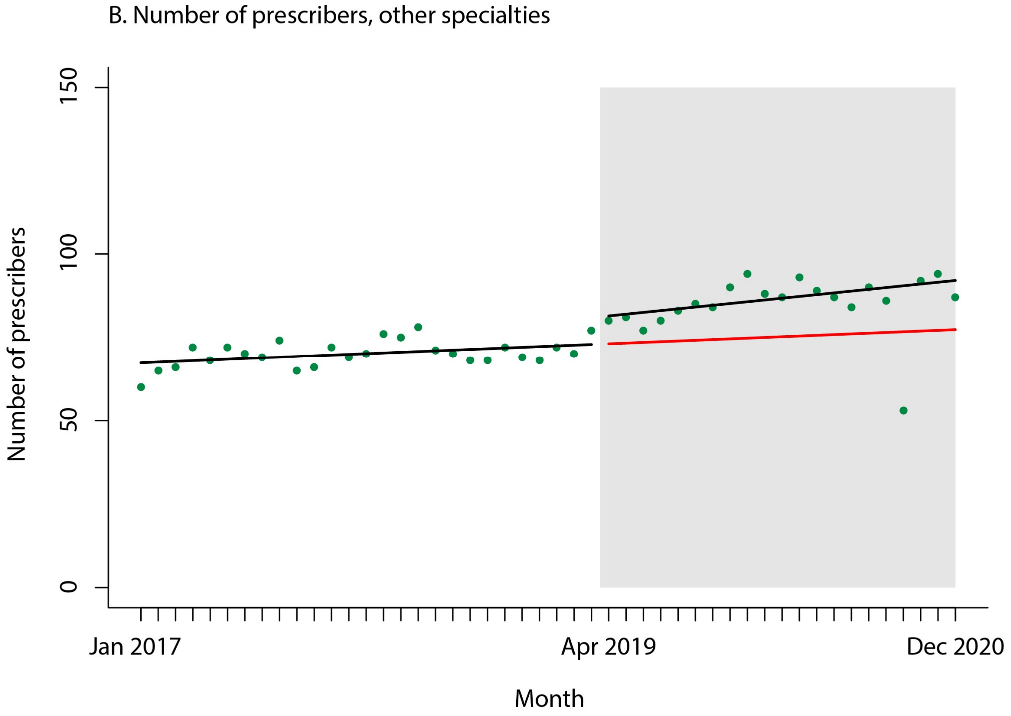


Other physician specialties include all specialties other than primary care, psychiatry, emergency medicine, and addiction medicine. Grey shaded area represents post-implementation period. Black line represents observed trends. Red line represents the counterfactual (i.e., projected post-initiative trends without implementation).

© 2023 Treitler P et al. *JAMA Network Open.*

**eFigure 7. Trends in the overall number of Medicaid buprenorphine prescribers before and after implementation of NJ Medicaid initiatives**

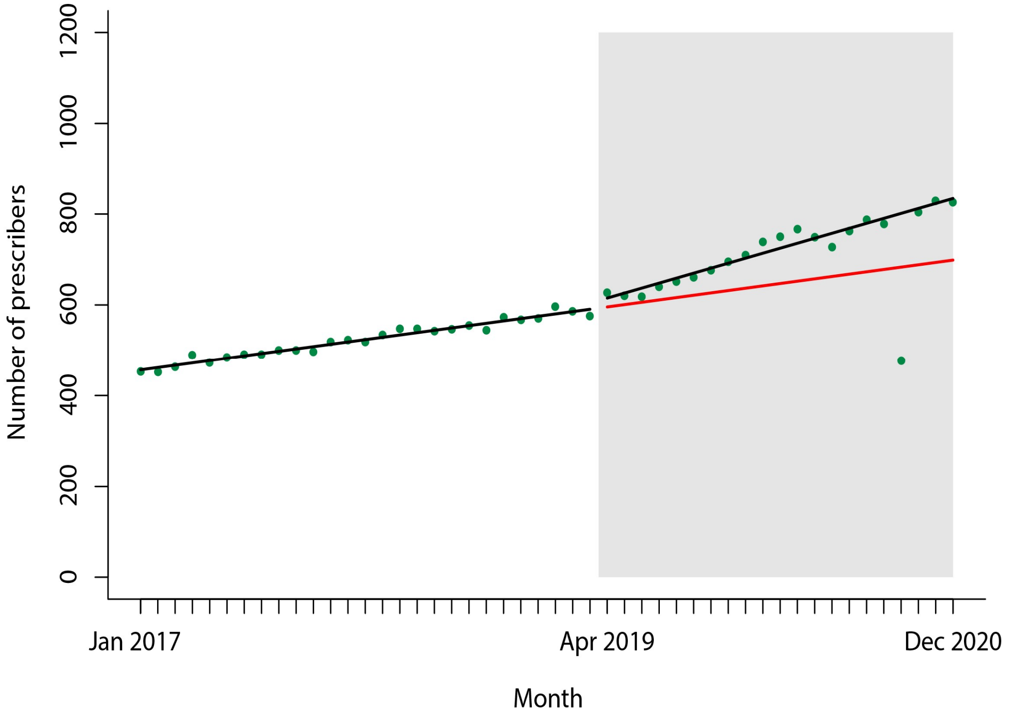


Grey shaded area represents post-implementation period. Black line represents observed trends. Red line represents the counterfactual (i.e., projected post-initiative trends without implementation).

© 2023 Treitler P et al. *JAMA Network Open.*

**eFigure 8. Trends in the number of Medicaid buprenorphine prescribers by provider type and specialty before and after implementation of NJ Medicaid initiatives**

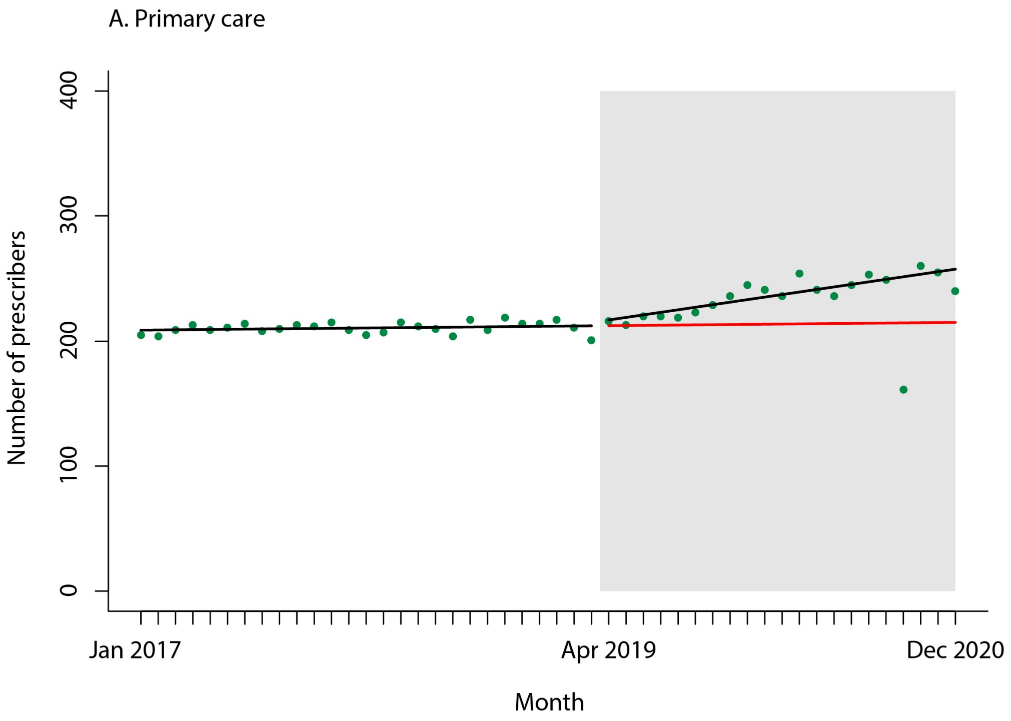


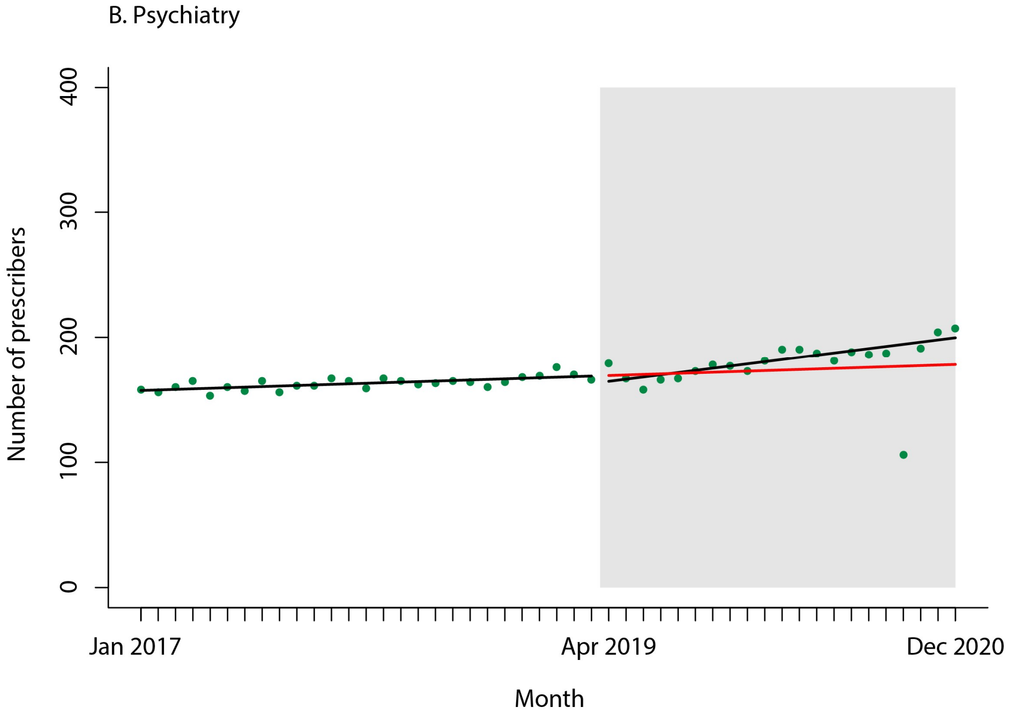


© 2023 Treitler P et al. *JAMA Network Open.*

C. Emergency medicine

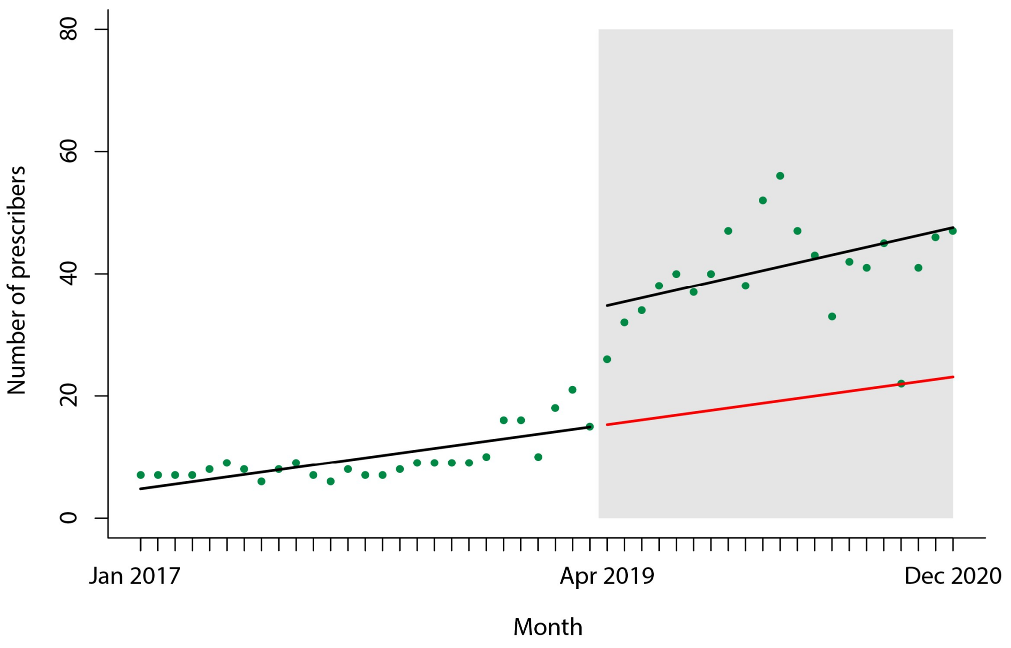


D. Advanced practitioners

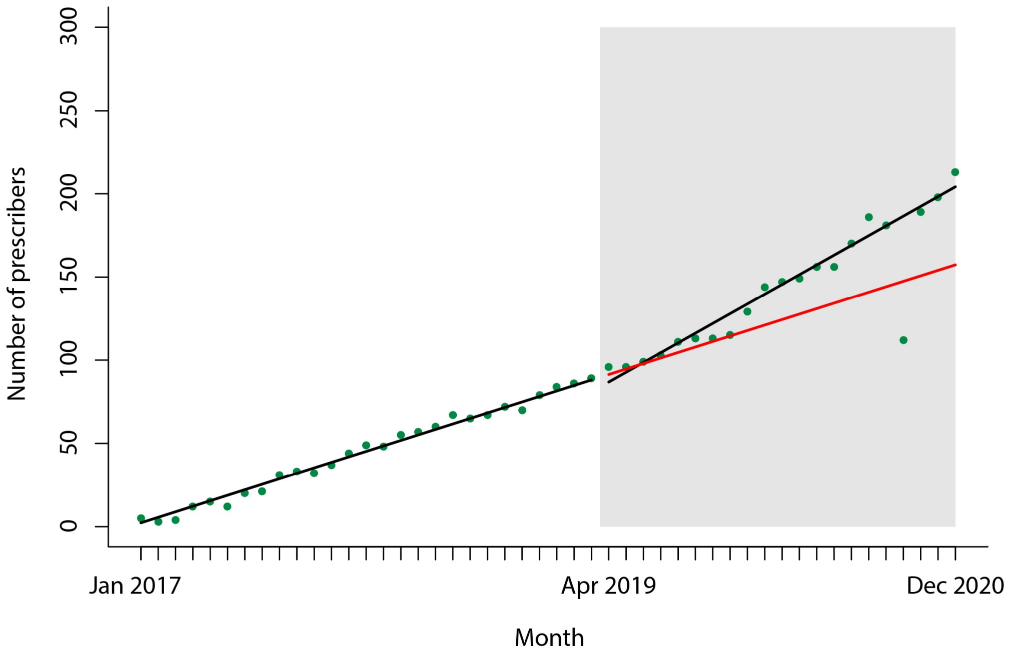


Grey shaded area represents post-implementation period. Black line represents observed trends. Red line represents the counterfactual (i.e., projected post-initiative trends without implementation).

© 2023 Treitler P et al. *JAMA Network Open.*

**eFigure 9. Trends in buprenorphine prescribers before and after implementation of NJ Medicaid initiatives, with washout period from January-March 2019**

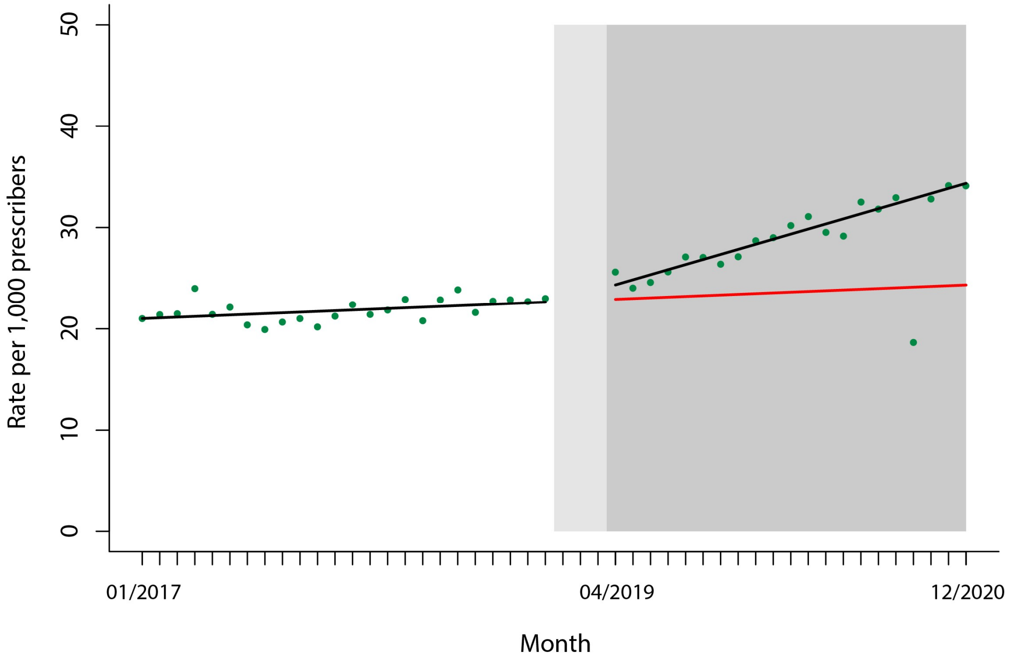


Grey shaded area represents post-implementation period. Black line represents observed trends. Red line represents the counterfactual (i.e., projected post-initiative trends without implementation).

© 2023 Treitler P et al. *JAMA Network Open.*

**eFigure 10. Trends in buprenorphine prescribers before and after implementation of NJ Medicaid initiatives, rate per 1000 Medicaid beneficiaries with OUD**

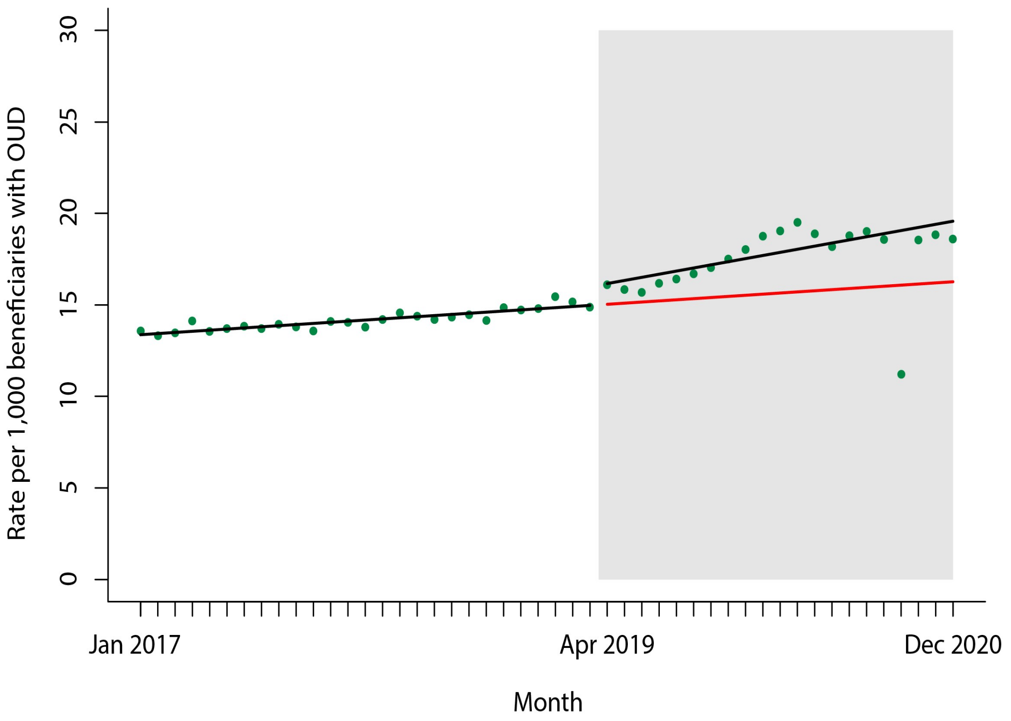


Grey shaded area represents post-implementation period. Black line represents observed trends. Red line represents the counterfactual (i.e., projected post-initiative trends without implementation).

© 2023 Treitler P et al. *JAMA Network Open.*

**eFigure 11. Trends in naltrexone prescribing rates per 1000 total Medicaid prescribers before and after implementation of NJ Medicaid initiatives**

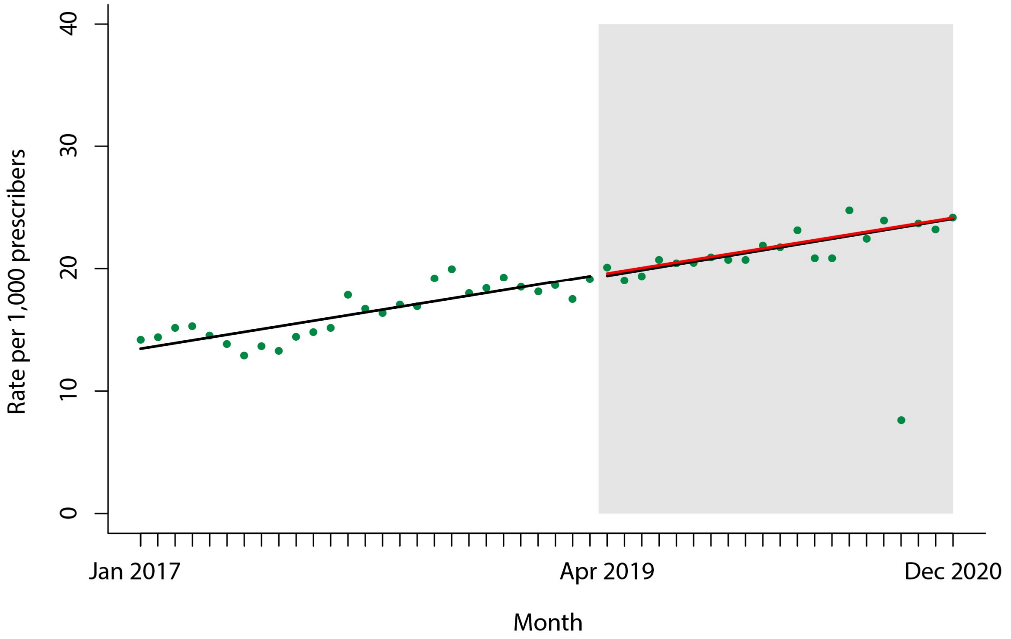


Grey shaded area represents post-implementation period. Black line represents observed trends. Red line represents the counterfactual (i.e., projected post-initiative trends without implementation).

© 2023 Treitler P et al. *JAMA Network Open.*

**eFigure 12. Trends in percentage of advanced practitioner buprenorphine prescribers before and after implementation of NJ Medicaid initiatives**

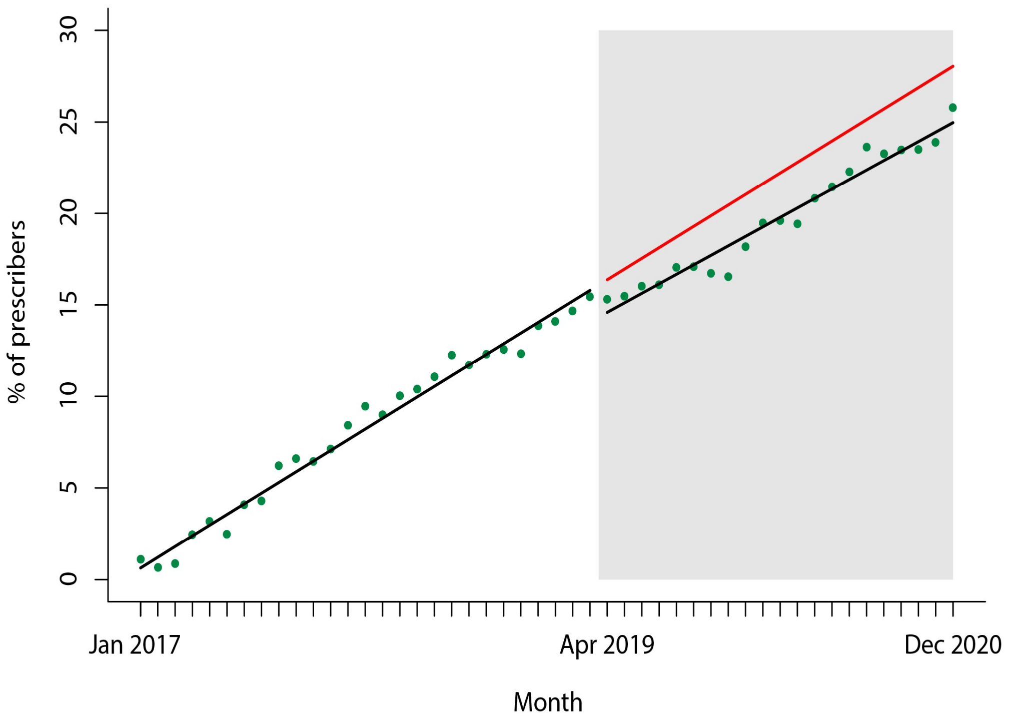


Grey shaded area represents post-implementation period. Black line represents observed trends. Red line represents the counterfactual (i.e., projected post-initiative trends without implementation).

© 2023 Treitler P et al. *JAMA Network Open.*

## eReferences


1. Mark TL, Dilonardo J, Vandivort R, Miller K. Psychiatric and medical comorbidities, associated pain, and health care utilization of patients prescribed buprenorphine. *J Subst Abuse Treat*. 2013;44(5):481-487. doi:10.1016/j.jsat.2012.11.004

2. Gordon AJ, Lo-Ciganic WH, Cochran G, et al. Patterns and Quality of Buprenorphine Opioid Agonist Treatment in a Large Medicaid Program. *J Addict Med*. 2015;9(6):470-477. doi:10.1097/ADM.0000000000000164

3. Lopez Bernal J, Cummins S, Gasparrini A. The use of controls in interrupted time series studies of public health interventions. International Journal of Epidemiology. 2018;47(6):2082-2093. doi:10.1093/ije/dyy135

4. Centers for Medicare & Medicaid Services. State Drug Utilization Data | Medicaid. Published December 19, 2022. Accessed March 9, 2023. https://www.medicaid.gov/medicaid/prescription-drugs/state-drug-utilization-data/index.html

5. Kaiser Family Foundation. Total Monthly Medicaid & CHIP Enrollment and Pre-ACA Enrollment. KFF. Published January 31, 2023. Accessed March 9, 2023. https://www.kff.org/health-reform/state-indicator/total-monthly-medicaid-and-chip-enrollment/


© 2023 Treitler P et al. *JAMA Network Open.*
