# Supplementary material for: Buprenorphine Utilization and Prescribing Among New Jersey Medicaid Beneficiaries After Adoption of Initiatives Designed to Improve Treatment Access
Source: JAMA Netw Open. 2023 May 5;6(5):e2312030. doi: 10.1001/jamanetworkopen.2023.12030 (PMC10163388; doi:10.1001/jamanetworkopen.2023.12030)
Supplement: Supplement 2. — Data Sharing Statement [file jamanetwopen-e2312030-s002.pdf]

Data Sharing Statement

Treitler. Buprenorphine Utilization and Prescribing Among New Jersey Medicaid Beneficiaries After Adoption of Initiatives Designed to Improve Treatment Access. *JAMA Netw Open*. Published May 05, 2023. doi:10.1001/jamanetworkopen.2023.12030

Data
Data available: No

Additional Information
Explanation for why data not available: The data that support the findings of this study are available from the New Jersey Department of Human Services. Restrictions apply to the availability of these data, which were used under a data use agreement for this study.
